# The Hebb Synapse Before Hebb: Theories of Synaptic Function in Learning and Memory Before Hebb (1949), With a Discussion of the Long-Lost Synaptic Theory of William McDougall

**DOI:** 10.3389/fnbeh.2021.732195

**Published:** 2021-10-21

**Authors:** Richard E. Brown, Thaddeus W. B. Bligh, Jessica F. Garden

**Affiliations:** Department of Psychology and Neuroscience, Dalhousie University, Halifax, NS, Canada

**Keywords:** engram, synaptic theory, cell assembly, ideas before their time, history

## Abstract

Since the work of Semon was rediscovered by Schacter in 1978, there has been a renewed interest is searching for the “engram” as the locus of memory in the brain and Hebb’s cell assembly has been equated with Semon’s engram. There have been many theories of memory involving some concept of synaptic change, culminating in the “Hebb Synapse” theory in 1949. However, Hebb said that the idea that any two cells or systems of cells that are repeatedly active at the same time will tend to become “associated,” was not his idea, but an old one. In this manuscript we give an overview of some of the theories of the neural basis of learning and memory before Hebb and describe the synaptic theory of William McDougall, which appears to have been an idea ahead of its time; so far ahead of its time that it was completely ignored by his contemporaries. We conclude by examining some critiques of McDougall’s theory of inhibition and with a short discussion on the fate of neuroscientists whose ideas were neglected when first presented but were accepted as important many decades later.

What has been will be again,
what has been done will be done again;
there is nothing new under the sun.

(Ecclesiastes 1:9)

## Introduction

What is the neural basis of learning and memory? This question has concerned philosophers, physiologists, and psychologists since ancient times (Burnham, 1888, 1889a,b; Yates, 1966). Following the development of the neuron theory and naming the synapse in the 1890’s there have been many theories concerning synaptic change, culminating in the “Hebb Synapse” theory (Hebb, 1949), but what were these theories? Hebb said that “The general idea is an old one, that any two cells or systems of cells that are repeatedly active at the same time will tend to become ‘associated’ so that activity in one facilitates activity in the other” (Hebb, 1949, p. 70). In this manuscript we give an overview of the pre-synaptic theories of the neural basis of learning and memory (before 1897), particularly those of James (1890) and the post-synaptic theories (between 1897 and 1949). Many of these theories are discussed by Lashley (1934); Hilgard and Marquis (1940), and Morgan (1943), but we focus on the synaptic theory of William McDougall, which appears to have been an idea ahead of its time; so far ahead of its time that it was completely ignored.

## The Modern Concept of the Engram as the Locus of Memory in the Brain

Although the term “engram” coined by Semon (1921) had a brief era of popularity early in the 20th century, it was mostly neglected until Lashley resurrected it in 1950 and then it occurred sporadically with reference to Lashley, and without reference to Semon (see Thompson, 1976) until early in the 21st century, when the engram of Semon became associated with the memory trace (Hübener and Bonhoeffer, 2010; Josselyn et al., 2015, 2017; Poo et al., 2016). The search for the engram has since taken on mythical proportions, with engram cells and engram circuits encoding memories that are not in non-engram cells (Langille and Gallistel, 2020). Since the work of Semon (1921) was “rediscovered” by Schacter et al. (1978) and Schacter, 2001, there has been a renewed interest is searching for the “engram” as the locus of memory in the brain. Devan et al. (2018) discussed “the emerging engram,” which is the topic of this special issue, however, Takamiya et al. (2020, p. 24) equated the concept of the “engram” with Hebb (1949) cell assemblies and say that “Populations of engram cells could be considered as cell assemblies encoding memory engrams.” While the location of a memory engram in the brain was vaguely defined by Semon (1921, pp. 120–123) as “the whole tract through which the synchronous excitation flows from its start to its cessation, whether through nerve-cells, nerve-fibers, the gray matter of the brain, or through other forms of irritable substance” (p. 121), the cell assembly is a mechanism for the formation of the memory trace and the modification of memory traces. Takamiya et al. (2020, p. 24) say that the “synchronous firing of neurons to encode a memory consists of a cell assembly being defined as functionally connected neurons via synchronous firing.” Thus, Hebb’s concept of synaptic change forms a cell assembly, which creates a long-term memory (Abraham et al., 2019), and this constitutes the “engram.” In other words, the cell assembly creates the engram.

In their glossary, Langille and Gallistel (2020) define the “Engram cell” as “A cell that is activated during learning and contains part of an engram.” The “Engram Circuit” is defined as “An ensemble of engram cells, which collectively embody the cellular change associated with something learned.” Finally, “Pattern Completion” is defined as “A process for activating an entire engram circuit from the activation of a subset of engram cells.” Figure 2 of their paper indicates that engram circuits and pattern completion are the result of synaptic change. In effect, the engram theory is simply a restatement of Hebb (1949) theory of learning and memory which encompassed the Hebb synapse, the Hebb cell assembly and the Hebb phase sequence. Synaptic plasticity is the fundamental event in learning and memory and the Hebb synapse is one of the theoretical mechanisms by which learning and memory occur (Keck et al., 2017; Langille and Brown, 2018; Abraham et al., 2019; Magee and Grienberger, 2020). Synaptic function is not only essential for learning and memory (Lisman et al., 2018) but synaptic dysfunction underlies both neurodevelopmental and neurodegenerative disorders of cognitive function (Taoufik et al., 2018; Batool et al., 2019). For a further discussion of the relationship between the engram and Hebb’s theory see Brown (2020).

## Donald O. Hebb’s Theory of Learning and Memory: The “Hebb Synapse; Cell Assembly and Phase Sequence

In *The Organization of* Behavior (1949), Hebb proposed that the neural basis of learning, memory and other psychological processes involved synaptic changes, cell assemblies and phase sequences, which connect the neurophysiological mechanisms studied by physiologists to thought and “mind” as studied by psychologists. While the Hebb synapse has become the most cited, and “better known than Donald Hebb himself” (Sejnowski, 2003), the cell assembly may be his most lasting legacy (Huyck and Passmore, 2013; Li et al., 2016; Poo et al., 2016; Eichenbaum, 2018; Sakurai et al., 2018). Hebb’s neurophysiological postulate, which defines the “Hebb synapse” (Hebb, 1949, p. 62) states that:

When an axon of cell A is near enough to excite a cell B and repeatedly or persistently takes part in firing it, some growth process or metabolic change takes place in one or both cells such that A’s efficiency, as one of the cells firing B, is increased.

The “**cell assembly**” (Hebb, 1949, pp. 69–74) was defined as a set of neurons and their connecting pathways which act together, such that the stimulation of one pathway will activate a reverberating circuit involving many connected pathways. This extended period of excitation bridged the gap between stimulus and response (Hebb, 1972, pp. 295, 304). A number of cell assemblies connected by patterned neural activity over time was defined as a “**phase sequence**” which provided the basis for a “train of thought” connecting cell assemblies (Hebb, 1949, pp. 79–106). The interactions among these three concepts give rise to the common phrase “neurons that fire together, wire together,” which is to say that neural pathways consistently activated together become physiologically modified to facilitate future signal transductions.

## Hebb’s Comments on the Origin of the “Hebb Synapse”

There have been many discussions about the Hebb synapse (Spatz, 1996; Kolb, 2003; Milner, 2003; Sejnowski, 2003; Cooper, 2005; Shepherd, 2010; Sweatt, 2016; Langille and Brown, 2018; Nadel and Maurer, 2020). However, the idea of the “Hebb synapse” was not new to Hebb and he did not feel that he was writing anything that had not been said before (Brown and Milner, 2003). He was compiling other people’s thoughts and postulates, and editing them so they could all work together as one clear explanation as to the physiological basis of learning. As noted by Hebb (1949, p. 70), “The general idea is an old one, that any two cells or systems of cells that are repeatedly active at the same time will tend to become ‘associated’ so that activity in one facilitates activity in the other.” Earlier papers have discussed how Hebb developed the theory that was presented in 1949 (see Brown and Milner, 2003; Brown, 2017, 2020) and this manuscript examines some of the theories that pre-dated Hebb’s synaptic theory. In researching this manuscript, we discovered the synapse theory of William McDougall, which seems to have been ignored by his contemporaries, so we summarize it here, as an example of an idea before its time (see Gross, 2009).

## Cell Theory, Neuron Theory, the Synapse and Localization of Function

The development of synaptic theories of the neural basis of learning and memory depended on cell theory, neuron theory, the concept of the synapse and the localization of function.

### Cell Theory

Although the ancient Greeks had proposed that matter was made of smaller parts (the atomic theory of Democritus, 430 BC), it was not until the invention of the microscope in the 17th century that the cellular basis of matter could be seen. In his 1665 volume entitled “*Micrographia*,” Robert Hooke (1635–1702) used the term “cell” to describe the microscopic units that he observed in cork (Hooke, 1665). Although others observed the cellular nature of plants and animals (Romero, 2011; Cocquyt et al., 2021), Theodore Schwann (1810–1882) and Mattias Jacob Schleiden (1804–1881) are given credit for founding “cell theory” in 1839 (Wolpert, 1995; Ribatti, 2018). This theory stated that the tissues of plants and animals are composed of individual cells, and each cell has a nucleus, intracellular cellular fluid and a cell wall. Among his many contributions to physiology, it was Jan Evangelista Purkyně (1787–1869) who discovered Purkinje cells in the cerebellum in 1837 (Cavero et al., 2017, p. 536).

### Neuron Theory Versus Reticular Theory

By 1863 **Otto Friedrich Karl Deiters (1834–1863)** had provided a detailed description of the nerve cell, identified the axon and dendrites and postulated that these nerve processes formed a continuous nerve network (Deiters and Guillery, 2013). Deiters called the axon an “axis cylinder” and the dendrites “protoplasmic processes,” and in 1871, **Joseph von Gerlach (1820–1896)** proposed that the brain was composed of a “protoplasmic network” (Weyers, 2020). According to Gerlach, the nervous system consisted of a single continuous network called the reticulum, and his work provided the impetus for the reticular theory “which postulated that all cells in the central nervous system were joined together as in an electrically distributed network” (Stahnisch, 2015). In 1873 Camillo Golgi (1843–1926) invented a new method for staining nerve cells for microscopic research and using this “black reaction,” he produced detailed descriptions of nerve cells in the cerebellum, cortex and olfactory bulb, showing individual neurons in great detail and distinguishing axons from dendrites (Pannese, 1999; Shepherd et al., 2011). By 1885, Golgi had developed his theory that the nervous system was an intricate network of intertwined branches of axons coming from different cell layers (a “diffuse nervous network”) connected by electrical nervous impulses (Shepherd, 2016). This formed the basis of Golgi’s reticular theory that neurons were connected by a network of nerve fibers, rather than a series of discrete cells (Cimino, 1999; Kruger and Otis, 2007; Raviola and Mazzarello, 2011).

Using Golgi’s black reaction, **Santigo Ramón y Cajal (1852–1934**) demonstrated that “the nervous system of vertebrates was comprised of billions of independent interconnected elements that are organized into neural networks” (Serrano-Castro and Garcia-Torrecillas, 2012, p. 1). Cajal’s diagrams of neurons in embryonic tissue using Golgi’s stain, published in 1888, were the first to show that “the terminal ramifications of neurons ended in arborizations that juxtaposed the body and the dendrites of other neurons without establishing continuity with them” (Serrano-Castro and Garcia-Torrecillas, 2012, p. 3). This is considered as the founding study of **the neuron doctrine** (Bock, 2013). In 1892, Cajal proposed his “neurotrophic theory” that stated that cells were attracted to one another through the secretion of chemotactic substances, and in 1894, he defined the “plasticity” of the nervous system. Cajal theorized that the brain structures were perpetually changing through their dendritic spines, which he considered the mechanism through which nerve cells adapted to their environment (Serrano-Castro and Garcia-Torrecillas, 2012, p. 4). Cajal further proposed “brain gymnastics” as a “mechanism for multiplying nerve connections and thus improving the brain’s functionality” (Serrano-Castro and Garcia-Torrecillas, 2012, p. 4). In 1895, Cajal proposed that glial cells could be involved in regulating the structural changes in dendritic spines and thus regulate neuronal activity.

In 1891, the German anatomist **Heinrich Wilhelm Gottfried von Waldeyer-Hartz** (1836–1921) wrote a series of six articles which summarized the work of Gerlach, Albert von Kölliker (1817–1905), Golgi, Cajal, and others on the microscopic anatomy of the nervous system, and in the last of these papers he coined the term “neuronen” as follows:

The nervous system is comprised of countless, connected anatomical and genetic neurons. Each neuron has three parts – the nerve cell, the nerve fibers and the fiber tree (end tree). The physiological conduction process can travel both in the direction from the cell to the fiber tree as well as vice versa. The motor conduction process travels only in the direction of the cell to the fiber trees, which are sensitive to initially one direction and then the other direction (Waldeyer, 1891, part 6, pp. 1352, 1353).

Thus, the **neuron doctrine** states that the nervous system is composed of discrete cells, a discovery based on the neuro-anatomical work of **Santiago Ramón y Cajal** but presented by **Waldeyer**, who coined the term ***neuron* (or *neurone*)** as a way of describing the basic structural units of the nervous system (see Winkelmann, 2007). The *neuron doctrine* considered neurons as special cases under **cell theory** (Guillery, 2005, 2007).

Before Golgi’s silver nitrate staining method, individual nerve cells could not be “seen,” but he showed that neurons, their cell bodies axons and dendrites (which Golgi called “short and long extensions”) could be visualized. Based on his staining method, Golgi became a leading advocate of the reticular theory. However, by 1888, Cajal determined that the nervous system was composed of individual nerve cells which were not physically connected to one another, providing the foundation for the **neuron doctrine** (Hellman, 2001, p. 97). In 1906, Cajal and Golgi were awarded the Nobel Prize in Physiology or Medicine. Golgi persisted in arguing that nerves were connected together in a reticular net and argued against Cajal’s definition of individual neurons. In his 1906 Nobel Prize acceptance speech, Golgi focused on the reticular hypothesis and ignored Cajal’s work, and even after Cajal’s 1906 Nobel Prize speech, many people still did not believe the neurone doctrine (De Carlos and Borrell, 2007; Grant, 2007; de Castro, 2019).

### Sherrington and the Synapse

In 1894, Cajal gave the Croonian Lecture at the Royal Society in London, met Charles Sherrington, and visited Oxford and Cambridge, where he was awarded an honorary degree (De Carlos and Molnár, 2020). According to Smith (1996), this lecture was “a landmark paper in the history of neuroscience.” In this lecture, Cajal noted that “the connections established between the fibers and the nerve cells take place by means of contact, that is, with the help of genuine articulations” and went on to say that “the cells are polarized, that is, the nerve current always enters by way of the protoplasmic apparatus of the cellular body, and it leaves by the axis cylinder which transmits it to a new protoplasmic apparatus” (Smith, 1996, p. 45). Based on the work of Cajal, Sherrington coined the term “synapse” in 1897 to name the gap between the axon and dendrite of two neurons. How the name “synapse” came about is described by Smith (1996, pp. 45–47).

Although there is general agreement that the first use of the term “synapse” was by Sherrington (1897) in Foster’s Textbook of Physiology (see Foster and Sherrington, 1897; Tansey, 1997; Bennett, 1999), Sherrington had earlier used the term “synapse” in his Croonian Lecture given to the Royal Society on 1 April 1897 (Sherrington, 1897, p. 221), and in his address to the British Association for the Advancement of Science meeting held in Toronto, Canada in August 1897, Sherrington wrote that “The place of linkage between nerve cell and nerve cell—the **synapsis** as it is termed by Professor Foster—is a place where the conduction of nervous impulses is supposed to occur across an intervening substance.” (Sherrington, 1898, p. 516). Therefore, the term “synapse” was in use as early as 1 April 1897 (see Black, 1981).

One of Sherrington’s main interests was measuring the time taken by a nerve stimulus to cross the synapse. Sherrington (1905, p. 740) stated that the waning of the scratch reflex of the spinal dog due to “fatigue” occurs at the synapse and he proposed that the delay in a reflex arc was due to the time taken for a signal to cross the synapse (Sherrington, 1906a). If that delay could be measured, one could calculate the number of synapses in a neural circuit. This was discussed by Schäfer (1900, p. 608) who said that:

A nervous path which includes any of the higher nerve centers or any complex nerve processes, must have a chain of several cells, with a synapse at the place of contact between each two links in the chain. There is reason to believe that the additional delay (“lost time”), which is characteristic of the passage of nervous impulses through the nerve centers, is due to a block at each synapse; that, in fact, the nervous impulses are momentarily arrested at these places of contact of the nerve-cells with one another. And it is not improbable that the relative number of these blocks will furnish a key to the differences which are found to obtain in the reaction time for different reflexes and psychical processes.

In *The Integrative Action of the Nervous System*, Sherrington (1906b, p. 21) determined that the speed of nervous transmission showed a delay at the synapse and in **1908**, **Florence Buchanan** published her work on the time taken for the transmission of reflex impulses in the spinal cord of the frog. After a series of experiments, she concluded that, although synapses had variable resistance, “That in the same-limb reflex there is normally a single synapse interposed in the conductive path of each individual fiber concerned, and that the time taken to pass it in the normal animal probably lies between 0.010 and 0.020 s” (Buchanan, 1908, p. 2).

### Localization of Function in the Brain

Another controversy which is relevant to early conceptions of the synaptic theory of learning and memory is that of the localization of function in the brain. In 1824, Pierre Flourens (1794–1867) proposed his principle of equivalence of structure and of mass action in the cerebral cortex. Flourens emphasized the integration of the nervous system and stated that: “although all of the various parts of the nervous system have specific properties, proper functions, distinct effects, and in spite of this marvelous diversity they constitute nevertheless a unified system. When one point in the nervous system becomes excited, it excites all others; one point irritated, irritates all. There is community of reaction. Unity is the great reigning principle” (Flourens, 1824, as cited by Tizard, 1959, p. 133). Flourens argued that psychological processes could not be localized, since they are simply aspects of a unitary spirit (see Zola-Morgan, 1995).

While the studies of Broca (1861); Fritsch and Hitzig (1870), Ferrier (1876) and later Sherrington and Grünbaum (1902) supported the concept of localization of function within the cortex, **Friedrich Leopold Goltz (1834–1902)** was critical of the concept of localization of function and supported the theory of equipotentiality. The Goltz-Ferrier debate of 1881 seemed to decide the issue in favor of localization of function (Tyler and Malessa, 2000), but there were those who did not believe in localization of function and who considered the cortex as equipotential. In his localization of the engram in the brain, Semon (1921) trod a fine line between supporting the concept of localization of function and equipotentiality. Responding to the histological studies of Brodmann (1909) who showed regional differences in the histological structure of the cortex, Franz (1912), labeled these findings as “the new phrenology,” and concluded that, “Notwithstanding our ignorance, it would appear best and most scientific that we should not adhere to any of the phrenological systems, however, scientific they may appear on the surface.” It is, therefore, not surprising that Franz’s student, Karl Lashley believed in the **mass action principle** rather than localization of function. Lashley’s research was an attempt to find the location in the brain responsible for learning and memory traces, a hypothetical structure he called **the engram** (Bruce, 2001). Lashley (1924) was opposed to the synaptic theory of memory and, based on his lesion studies with rats learning mazes, Lashley (1929) concluded that the proportion of the brain that was lesioned was directly proportional to the decreased memory function, and that memories could not be localized in a single cortical area but were distributed throughout the cortex. In this way, Lashley supported the theory of equipotentiality in the cortex and opposed the theory of the localization of function.

## Theories of the Neural Location of Memory Before the Synapse (1897)

As pointed out by Hebb, there is a long history of theories about the neural basis of learning and memory. Many of these theories were proposed before the advent of neuron theory and the naming of the synapse in the 1890’s, and so were general theories about the neural basis of mental functions. Prior to the 1800’s, theories of memory and learning were developed by philosophers including Plato, Aristotle, and Descartes. Plato claimed that the mind was akin to a wax tablet, with perceptions leaving lasting impressions on the mind and believed that “when the wax in one’s soul is deep, abundant, smooth, and of the right quality, the impressions are lasting. Such minds learn easily, retain easily, and are not liable to confusion” (Burnham, 1888, pp. 41, 42). By the late, 19th century, most physiological and pathological studies pointed to a physical process underlying memory: “A cerebral process of some kind is the physical concomitant of an idea, and the condition of the reproduction of the idea is the repetition of the original cerebral process” (Burnham, 1889a, p. 569).

Early proponents advocated three different theories for the physical basis of memory: (1) a *movement* persisting in the brain; (2) a persisting *trace* in the brain; or (3) a *disposition* persisting in the brain. The theory that memory involved a persistent movement in the brain stated that: “vibrations of an impression persist in the nervous elements, and to revive the impression it is only necessary that a suitable awakening agency renew the vibrations” (Burnham, 1889a, p. 571). The theory that memory depends on a persisting trace, gathered significant support from both Charles Robert Richet (1850–1935), who contrasted the effects of stimulation on muscle and on nerve cells, concluding that “while the muscles and the organic nerve-cell return completely to their original condition after an excitation, the psychic nerve-cell does not. It has been modified in a permanent manner by the act of stimulation” (Burnham, 1889b, p. 573). The theory that memory is a *disposition* persisting in the brain was supported by Wilhelm Wundt (1832–1920) and Theodule-Armand Ribot (1839–1916). According to Ribot, the basis of memory lies “not only in the modifications effected in the individual elements, but also in the way various elements are grouped together to form a complex” (Burnham, 1889b, p. 574). Thus, Ribot and others stressed the importance of complex nerve associations, comparable to the engram and to Hebb’s cell assembly theory. Although Burnham (1889b) described how each of these three theories were related, in 1889 there was no knowledge of the microscopic anatomy of the brain, nor of the functional connections between neurons.

### William James (1890)

William James (1842–1910) completed an MD at Harvard Medical School in 1869 and spent a year (1867–1868) traveling in Germany, where he became interested in philosophy and psychology. He was appointed instructor in physiology at Harvard in 1872 and assistant professor of psychology in 1876 and remained at Harvard until he retired in 1907. James became influential for his book, *The Principles of Psychology* (1890), which was 1,200 pages long, published in in two volumes and took him 12 years to complete. Although this book was written before neuron theory and the naming of the synapse, it became a standard textbook in Psychology for over 30 years. The theories of William James on the laws of habit, association, and memory were used by McDougall and by Hebb to develop their ideas of the neural basis of perception and memory. Berlucchi and Buchtel (2009) credit James with using the term “*plasticity*” to denote changes in the nervous pathways associated with the formation of habits as he proposed that “the phenomena of habit in living beings [is] due to the plasticity of the organic materials of which their bodies are composed” (James, 1890, p. 105).

In *The Principles of Psychology*, James (1890, pp. 24–26) focused on the problem of how physiological processes in the brain become organized so as to “*correspond to reminiscences in the mind.”* He then described his theories on the neural basis of consciousness, the stream of thought, attention, perception, reasoning, and the emotions. Early in the book, he defined his “law of association” by stating that:

The same cerebral process which, when aroused from without by a sense- organ, gives the perception of an object, will give an *idea* of the same object when aroused by other cerebral processes from within. If processes 1, 2, 3, and 4 have once been aroused together or in immediate succession, any subsequent arousal of any one of them (whether from without or within) will tend to arouse the others in the original order [This is the so-called law of association] (p. 24).

James also advocated the idea that neural components which become active concurrently can merge into each other, forming new neural connections, which he described as his “law of mental association by contiguity” as follows:

Objects once experienced together tend to become associated in the imagination, so that when any one of them is thought of, the others are likely to be thought of also, in the same order of sequence or coexistence as before. This statement we may name the law of mental association by contiguity (James, 1880, 1890, p. 561).

In a footnote, James (1890, p. 561) quotes earlier versions of the law of mental association by continuity by David Hartley (1705–1757) and Alexander Bain (1818–1903) and proposes a physiological basis for this law, which he terms “the law of neural habit” (p. 564):

When two elementary brain-processes have been active together or in immediate succession, one of them, on reoccurring, tends to propagate its excitement into the other (p. 566).

## Three NearLy-Synaptic Theories of Memory

Three theories for the neural basis of learning and memory that came close to Hebb’s synaptic theory were proposed in the late 1890’s by Tanzi, Freud, and Pavlov.

### Eugenio Tanzi (1893)

According to Berlucchi and Buchtel (2009), Eugenio Tanzi (1856–1934) first proposed that associative memories depend on a localized facilitation of neural transmission at the junctions between neurons in 1893, 4 years before the term “synapse” was coined. At that time the diffuse nerve net theory of Golgi was predominant, however, Tanzi and his student Ernesto Lugaro (1870–1940) supported the neuron doctrine of Cajal and proposed that nervous excitation must encounter some resistance in crossing the nerve junctions. Tanzi proposed that the repetitive activity in a neural path during learning could cause “a hypertrophy of neurons along that path, thus reducing the distance between neurons and making the crossing easier.” Lugaro (1909) used the term “plasticity” for experience-related synaptic changes. He also proposed a “chemotropism” between neurons, based on Cajal’s concept of neural growth cones. Lugaro (1909) also presented a theory that mental associations could depend on associations between neurons, based on a coincidence of activity, similar to that proposed by William James (see Berlucchi and Buchtel, 2009).

### Sigmund Freud (1895)

Sigmund Freud (1856–1939) is best known for his psychoanalytic theories; however, his early publications focused on neurobiology and neurology. In his theoretical treatise, “*Project for a Scientific Psychology*,” written in 1895 and published posthumously, Freud (1895/1953) proposed that the resistance between neurons at locations called “contact barriers” could change so that some of these contact points would allow the passage of excitation easily, while others might do so partially or with difficulty. It was these contact points or junctions between neurons that Sherrington named synapses in 1897. In a convergence of ideas with Cajal, Freud postulated that learning might produce prolonged changes in the effectiveness of the connections between neurons and that such changes could serve as the mechanism for memory (see Triarhou and del Cerro, 1985).

### Ivan Pavlov (1903)

In 1903, Ivan **Pavlov (1849–1936)** proposed a neural mechanism underlying the conditioned reflex and stated that when the “psychical” stimuli associated with the food:

become connected with the same nervous center of the salivary glands to which the stimulation emanating from the essential properties of the object is conducted through a fixed centripetal path. It can be assumed in this case that the salivary center acts in the central nervous system as a point of attraction for stimuli coming from other sensory surfaces. Thus a certain path is opened from the other excited areas of the body to the salivary center. But this connection of the center with accidental points is very fragile and tends to disappear of itself. Constant repetition of simultaneous stimulation by means of the essential and unessential properties of the object is required to make this connection increasingly durable. **(Pavlov**, **1903/1955**, **p**. **163**).

Thus, Pavlov’s theory of the neural basis of conditioning sounds very similar to that of William James and to the “Hebb synapse,” but Pavlov was concerned with providing a physiological rather than a psychological explanation for the conditioned response and he was critical of psychological (introspective) theories. He said that:

The investigation of the conditioned reflexes is of very great importance for the physiology of the higher parts of the central nervous system. Hitherto this department of physiology has throughout most of its extent availed itself of ideas not its own, ideas borrowed from psychology, but now there is a possibility of it being liberated from such evil influences (Pavlov, 1906, p. 915).

These three theories all proposed that changes in the connections between neurons at the nerve junctions provided a neural basis for learning and memory, however, McDougall proposed a synaptic theory which included a chemical signal at the synapse which he felt accounted for many psychological phenomena, from attention to learning, memory, fatigue, sleep, and hypnosis.

## William Mcdougall’s Synaptic Theory: So Far Ahead of Its Time, It Was Completely Ignored

Sometimes one discovers a set of published papers that come “out of the blue” as a complete surprise. Such was the case of the publications of William McDougall between 1898 and 1906 on his synaptic theory of neural processes. Since this theory seems to have been completely ignored both during its time and subsequently, we describe it here in detail.

### Who Was William McDougall?

William McDougall (1871–1938) graduated with first class honors in biology from the University of Manchester in 1890, and then studied medicine at St John’s College, Cambridge specializing in physiology, anatomy, and anthropology. After graduating from Cambridge in 1894, he completed his medical degree at St Thomas’ Hospital, London, where he undertook research on the problem of muscular contraction in the Physiological Laboratory of Sherrington (McDougall, 1930). During this time, he was influenced by **William James’ *Principles of Psychology***, and decided to study the nervous system “from below upward by way of physiology and neurology, and from above downward by way of psychology, philosophy, and the various human sciences (McDougall, 1930, p. 200). He then went to Gottingen, Germany to study experimental laboratory methods in psychology under Georg Elias Müller (1850–1934) and carried out experiments on color-vision and on attention in Müller’s laboratory. Upon returning to England in 1900, McDougall became an instructor in psychology at University College, London where he continued his research on vision and on attention. Between 1898 and 1908, McDougall published a number of papers and a small book on physiological psychology. It was his conviction that mental processes “emerged from the complex conjunctions of brain-processes.” In these papers, McDougall expanded on the physiological theories of William James and added ideas about the “transfer of energy” from one cell to another through the junction between two cells, the synapse. He also proposed a chemical transmitter substance which he called “neurin” and a physiological mechanism for inhibition, which became known as his “drainage theory.” In his autobiographical chapter, he noted that:

As regards the general functioning of the brain, I could not accept the view then and still now current among the physiologists, namely, that each neuron merely transmits to its neighbors a stimulus. It seemed to me clear that the beginning of all understanding of brain-functioning was to regard the brain as the seat of action of fields of energy, within which fields there was widespread reciprocal influence and free flow of energy from part to part. In both my main interests, then, I was as usual opposed to the popular or orthodox views. In consequence, most of my contributions of that period have remained buried in their original depositories (McDougall, 1930, p. 206).

In 1904 McDougall was appointed to the Wilde Readership in Mental Philosophy at Oxford, where he lectured in “the whole field of psychology conceived in the broadest way.” This position stipulated that *“the reader should study the human mind by observation and reflection on experience, while excluding any form of experiment,”* but McDougall circumvented these restrictions, by securing rooms from Professor Francis Gotch (1853–1913) in the Physiology Department, where he carried out experimental research on color vision and perception with a small group of students. However, during his time at Oxford, he did not fit into any niche. He states that:

I was neither a scientist nor a philosopher *pur sang.* I fell between two stools. The scientists suspected me of being a metaphysician; and the philosophers regarded me as representing an impossible and non-existent branch of science. Psychology had no recognized place in the curricula and examinations (McDougall, 1930, p. 207).

McDougall (1908a) wrote his book on *Social Psychology (which went through 23 editions)*, and *Psychology, the Study of Behavior (McDougall, 1912)*, which sketched the scheme of the psychology developed in more detail in his *Outline of Psychology* (McDougall, 1923). In 1919, McDougall wrote *The Group Mind* (McDougall, 1920), but its reception was so unfavorable that his *magnum opus* on social psychology was neglected as he found it “increasingly difficult to believe in the value” of his work (McDougall, 1930, p. 212). Becoming increasingly frustrated at Oxford, McDougall accepted the William James Chair of Psychology at Harvard University in the summer of 1920 where he gave a series of lectures in Psychology and published his *Outline of Psychology* (1923) and *Abnormal Psychology* (McDougall, 1926a). However, he was not well received in the United States. As he himself noted, he was prone to champion ideas that were rejected by the mainstream in psychology and physiology and at Harvard he was a vocal critic of behaviorism, proposed that human behavior was based on a catalog of instincts, supported eugenics and set forth on a long series of experiments using white rats to study Lamarckian inheritance (McDougall, 1927, 1930; Rhine and McDougall, 1933). He came to believe that the main problem with psychology was the acceptance of mechanistic biology, and the neglect of the purposive or teleological aspect of mental life. He attempted to develop a “purposive psychology,” which focused on the purposive nature of mental activity, arguing that the most essential character of life-processes was their goal-seeking nature and that such goal seeking developed through evolution into specialized forms of instincts. He felt that mental activity, knowing, feeling, and striving, had an innate basis, and that the mind had a “relatively simple innate structure.” He noted that “In America I was known as a writer who had flourished in the later middle ages and had written out a list of alleged instincts of the human species” (McDougall, 1930, p. 216). In 1924, Watson and McDougall (1929) Watson had a public debate, which was published as *The Battle of Behaviorism*. At the same time, he continued to do research on psychic phenomena, and served as the president of the American Society for Psychical Research. McDougall moved to Duke University in 1927, where he established the Parapsychology Laboratory, edited the *Journal of Parapsychology* and published a book on *Extra Sensory Perception (Rhine and McDougall, 1934)*, which was co-authored by J. B. Rhine. McDougall died in Durham, North Carolina, in 1938.

### McDougall’s First Neural Theory of Psychological Processes: 1898

If one is only familiar with their later works, than McDougall, like Freud, seems the most unlikely person to have proposed a neural theory of psychological functions. But he did. McDougall (1905, pp. 142–145) used *The Principles of Psychology* by William James as the starting point for his theories of the neural basis of learning and memory. In his first series of three papers, McDougall’s aim was to “describe mental activity in terms of consciousness and nervous processes” (McDougall, 1898a, p. 23). His proposal was that “all or almost all psychical processes are in some way dependent upon neural processes” (McDougall, 1898a, p. 30). This idea led him to the conclusion that changes in the nervous system due to experience must occur at the junctions between neurons (The term synapse was not yet coined by Sherrington). Much of the first paper was a critique of how James Ward (1843–1925) defined *Psychology* in the ninth edition (Ward, 1886) of the Encyclopedia Britannica (Basile, 2017) and his neural theory of memory was outlined in the second paper, where he stated that: “if we try to express experience in terms of neural process we must, I think, say that it means the establishment of new relations among nerve cells and their processes, or using the word neuron as the name for each nerve cell and all its processes, we may say that it is the establishment of new connexions among neurons” (McDougall, 1898b, p. 164).

During the years that McDougall spent completing his medical qualifications at St Thomas’ Hospital in London (1894–1898) and working with Sherrington, he spent his summer vacations in the physiology department at Cambridge, where he wrote his essay on an improvement in the psychological method (in three parts) of which the first two parts were read before the Aristotelian Society on 29 November 1897. These essays were clearly influenced by the work of Sherrington on reflex arcs, muscle physiology and fatigue, the work of T. R. Elliot on adrenaline and J. N. Langley on nicotine and other drugs.

McDougall’s third paper in this series examined the relationship between neurons, the junctions between them, and “the more complex functions of the mind” (McDougall, 1898c). From this paper, it is clear that McDougall believed in the localization of function in the cerebral cortex and that “consciousness accompanies the process of establishment of new connexions among neurons” (p. 366). He then considered the neural basis of associative learning:

a system may establish inter-connexions with other systems, through their excitement contemporaneously, or in close proximity in time, i.e., by association by contiguity in time. For there are good reasons for believing that the physiological barriers of a system are not absolute; that when a system A is excited, the main current of the flow of impulses passes through the nerve-paths constituting that system, but if any other system B, is excited at the same time or immediately afterward, then the excitement has spread from A to B. This tract is then more permeable in the future, and any excitement of A tends to spread to B also, and conversely. Between the two systems A and B there is thus established a connexion, and they form now a single, more complex system, of which A and B are sub-systems. The motor re-action to which the excitement of this new system leads, is the resultant of the re-actions of the two systems (McDougall, 1898c, p. 367).

Here McDougall is saying, in effect, that cells that fire together wire together. Of course William James said this in 1890, but McDougall (1898c, p. 374) expanded this when he asserted that: “the essential condition of the occurrence of consciousness is the making of new nerve paths, the establishment of new functional connexions between neurons.” Consideration of the effects of drugs such as alcohol, curare and atropine upon consciousness led McDougall (1898c, p. 381) to conclude that:

it seems highly probable that fixity of functional continuity depends upon the intimacy of **the junctions** between processes of the neurons and that these drugs act chiefly upon the junctions tending to abolish their conductivity for the nervous impulse. This view of the action of the drugs would afford a very satisfactory explanation of their analgesic action.

The phenomena of fatigue seems to point to the same conclusion; for it has been experimentally shown that in excised tissues **the junction** between nerve and muscle is more readily affected by fatigue than either nerve or muscle.

This hypothesis finds further support in observations on the action of nicotine on the ganglia of the sympathetic nervous system. For it has been shown by Langley (1896) to be highly probable that nicotine abolishes **the functional continuity between neuron and neuron** in these ganglia, without markedly affecting the neurons themselves (McDougall, 1898c, pp. 381, 382).

### McDougall’s Revised Synaptic Theory of Psychological Processes: 1901

In his 1898 papers, McDougall showed that he believed in (1) the neuron doctrine of Cajal; (2) the localization of function and (3) that changes in the nervous system occurred at the junctions between neurons. McDougall then expanded on his neural theory of memory to include the recently named synapse and he proposed a theory of chemical communication between synapses. McDougall had considerable experience in the physiological psychology of his time: he had done a degree in physiology at Cambridge and worked with Sherrington at St Thomas’s hospital. He had studied with Müller in Gottingen and he worked at UCL, where Bayliss and Staring discovered hormones and where Sir Henry Dale and Otto Loewi studied acetylcholine, the first chemical transmitter. Thus, he must have picked up ideas from all of them in order to develop his theory.

After his years in Göttingen with Müller, McDougall taught psychology and physiology at University College, London (UCL) between 1900 and 1904. He did research on color vision (McDougall, 1901b) and on “the general functioning of the brain, the synaptic functions, inhibition, and the phenomenon of attention” (McDougall, 1930, p. 206). These papers, like his earlier papers, were influenced by the work of Sherrington and Langley, but he was also influenced by his colleagues in both psychology and physiology at UCL. Francis Galton (1822–1911) had established an Anthropometric Laboratory at UCL in 1889 and in 1898, James Sully (1842–1923), the Grote Professor of Mind and Logic, set up the psychological laboratory at UCL with the support of Francis Galton and the professors of physics and physiology (see Valentine, 1999). McDougall was hired by Sully to teach sensory psychology and on 24 October 1901, Sully, McDougall and eight others, including Sophie Bryant (1850–1927) and Frederick Walker Mott (1853–1926) founded the British Psychological Society which published the first issue of the British Journal of Psychology in 1904 (Edgell, 1947). McDougall taught a course on experimental laboratory methods while doing research on visual psychophysics in his home laboratory in two attic rooms in “a small house on the Surrey Downs near Haslemere” (McDougall, 1930, p. 205).

As well as contributing to the development of psychology at UCL, McDougall was involved in research and teaching in physiology. Like the Psychological Society, the panel of University Lecturers in Physiology and Experimental Psychology was also founded in 1901 and included McDougall teaching on the sense organs, and Mott on the Central Nervous System, as well as courses by W. M. Baylis (1860–1924), Ernest Henry Starling (1866–1927), Leonard Hill (1866–1952), and A. D. Waller (1856–1922). Sherrington and Rivers were also listed as faculty. Special lecture and laboratory rooms were provided for this course at UCL as diagramed in the British Medical Journal (Anonymous, 1902). Thus, McDougall rubbed shoulders with the leading psychologists and physiologists in England who were doing research on nerve activity and the action of drugs and chemical signals in the body. McDougall was at the epicenter of British psychology and physiology at a time when the physiology of the brain was a hot topic. It was in 1902 that Bayliss and Starling isolated the first hormone from the stomach of dogs which they called secretin (Bayliss and Starling, 1902; Modlin and Kidd, 2001). At Cambridge, Elliot was examining the functions of adrenaline (Elliott, 1904) and Langley was proposing the existence of receptors for chemical drugs such as nicotine, curare, and atropine (Langley, 1905). In fact, Langley was on the verge of proposing a chemical theory of neural communication, so the topic was “in the air” between 1900 and 1905 and McDougall seems to have been breathing that air. Unfortunately, while almost all of McDougall’s colleagues were mentioned in the history of physiology in Great Britain (Sharpey-Schafer, 1927, 1932), McDougall was not mentioned. However, the atmosphere in physiology at UCL and Cambridge at the time clearly influenced McDougall’s thinking about the neural control of psychological processes.

In his first paper of the twentieth century, McDougall (1901a) presented his theory on the role of the synapse in the physiology of the nervous system and in psychological functions. One might argue that McDougall (1901a) wrote the first ever paper which claimed that synaptic plasticity in the brain was the basis of thought and behavior. He stated (p. 582) that “those who adopt the spiritualistic hypothesis will have to regard **the synapses** as the places of interaction of soul and body.” Based on Schäfer (1900) chapter on the nerve cell, McDougall realized the importance of the synapse in neural conduction and that synapses had different levels of “resistance.” McDougall’s aim was to discover how a psychical event and its underlying physiological changes were related and to do this he examined anatomical, physiological, and psychological evidence. Under the **anatomical** evidence, McDougall reiterated his belief in the neuron theory and his belief that neurons were connected through synapses. This was his first use of the word “synapse” to describe the junction between two neurons. Under the **physiological** evidence, McDougall reviewed what was known about the synapse in 1901 in a series of five points:

(1) We know that there are various states of the central nervous system characterized by differences in the degree of resistance offered to the propagation of the nervous impulses from the afferent to the efferent nerve-fibers, and that the **synapses** are the seats of these changes in the degree of resistance.
(2) We know also that, in passing through the central nervous system, the impulses are delayed, there is occasioned in some way what is known as the “lost time.”
(3) Thirdly, we know that some part of the central conduction-path acts like a valve, permitting the passage of impulses from the afferent toward the efferent side, but preventing entirely, or at least offering great resistance to, passage in the reverse direction.
(4) Fourthly, we know that a very rapidly repeated or a continuous stimulus thrown into an afferent nerve or into the motor cortex issues in the efferent nerves as a regular series of impulses at the rate of about ten to nineteen per second, and that in voluntary effort also a similar series of impulses issues along the efferent nerves.
(5) It was also known that the synapses were the seats of the valve-effect and are therefore at the root of what was called the “law of forward-conduction of nerve-impulses,” and that they occasion the lost time of reflexes and simple reactions (McDougall, 1901a, pp. 586, 587).

McDougall then discussed whether changes in the nervous system occurred in the nerve cell body or in the synapse, an issue which is still with us, as indicated by the debate between Langille and Gallistel (2020). McDougall said that:

It is perhaps worthwhile briefly to resume the evidence that all these effects, the varying resistance, the lost time, the valve-like blocking of impulses, and the transformation of the continuous excitation, or rapidly repeated series of excitations, into a series of moderate rate, are determined at the **synapses** and not at the cell-bodies; for here again there seem to be only two possibilities—the problem appears as a dilemma—cell-body or synapsis (McDougall, 1901a, p. 587).

McDougall then provided evidence against the cell-body and for the synapse as the location of changes in resistance in the nervous system.

In evidence against the cell-body we have the following facts: firstly, it seems to be established that in many cases, if not in all, the fibrils of the cell processes run through the cell-bodies without interruption, and there seems to be no direct evidence of any sort to show that the bodies of cells offer any resistance to the passage of impulses through them; we know that in passing through a spinal ganglion the impulse suffers no appreciable time delay (see Schäfer, 1900. chapter on the “Nerve Cell,” in his *Text-book of Physiology*, p. 604), and that impulses, or at least electric changes such as always accompany a nerve-impulse, may be transmitted through the spinal ganglia in both directions [as found by Gotch and Horsley (1891)].

That the synapses are the seats of the valve-effect and are therefore at the root of what has been called the “law of forward-conduction of nerve-impulses,” and that they occasion the lost time of reflexes and simple reactions, are propositions now pretty generally accepted. The former seems to be proved by the experiments of Gotch and Horsley, which showed that the nerve-impulse may be transmitted in the reverse of the normal direction through the spinal ganglia and through the efferent nerves, but is not, in the latter case, passed on to other neurones of the cord; while the latter is rendered highly probable by the simple process of excluding the alternative, the cell-body, no lost time being demonstrable in the case of the spinal ganglia (McDougall, 1901a, pp. 587, 588).

McDougall argued that the “lost time” in reflex activity was due to the time taken for the transmission of excitation between neurons at synapses, as measured by Sherrington and reported in Schäfer (1900). He then argued that as neural pathways are used more frequently, the resistance at the synapses decreases, resulting in decreased reaction times.

We must believe that in the reflex conduction-paths of the cord, paths of a high degree of constancy of function, the synapses are very thoroughly organized, i.e., that their degree of resistance has been reduced to a minimum by frequent repetition of the particular reflex action, while in the higher parts of the nervous system the resistance, and therefore the loss of time, occasioned by the synapses is greater in the inverse order of their degree of organization.

This conclusion is borne out by the study of the effects of practice in shortening reaction-times. It is well known that the simple reaction-time may be somewhat shortened by practice until it becomes a purely automatic or reflex act, and in a similar manner reactions involving more complex mental processes, such as discrimination and choice, may on frequent repetition become more and more automatic in character, i.e., they are performed more easily and regularly and with less and less clear conscious accompaniment, until they, too, resemble rather a reflex action, psychical activity being reduced to a minimum. And parallel with this progressive loss of psychic accompaniment goes a progressive **shortening of the time lost at the synapses** (McDougall, 1901a, pp. 589–590).

McDougall then proposed the “resistance theory” of synaptic change, based on the observations of Langley and others on the effects of drugs on nervous conduction, and the work of Sherrington and others on “fatigue”:

This shortening of the lost time at the synapses by repetition of the passage of the excitation across them may be best conceived as due to a diminution of the resistance offered by that delicate and complex inter-cellular substance which, as it seems to me, we have to regard as the seat of the psycho-physical processes.

The junctions of nerve-fibers with the cells of glands and muscles are also peculiarly easily affected by drugs and by fatigue, and it is not improbable that the constitution of such junctions is similar to that of the junctions of the neurones.

This is just the series of effects that must result if these drugs act first and chiefly upon the inter-cellular substance at the synapses, slowing or paralyzing its metabolism in some degree and so raising its normal resistance. For where the resistance is normally greatest there complete blocking of the conduction path must earliest result, and where it is least, conductivity will persist longest, the synapses will remain longest permeable to the nervous impulse.

If, as I suggest, the fatigue is synaptic and results in an increase of the normal resistance of the synapses, then it is obvious that the passage of a weak excitation may be barred by a smaller degree of fatigue, a smaller increase of the normal resistance than would be necessary to bar the passage of a stronger excitation (McDougall, 1901a, pp. 591–593).

Under **the psychological evidence**, McDougall (1901a, pp. 605, 606) explained mental processes in terms of neurons and synapses and proposed a role of synaptic plasticity in cognitive function. He began by describing mental activity as a series of associations and then presented his hypothesis for the neural basis of mental functions. This involved synaptic changes, and the connections between specific sets of neurons which are organized into functional groups. When you read this, you can see the concepts of the Hebb synapse, cell assembly and phase sequence being thought out in 1901! McDougall (1901a) proposed that these neural changes represented “the physical basis of memory,” as described in his own words:

When we attempt to translate all this into terms of neural process, our account must run in some such way as follows: The mass of neurons constituting the nervous system is organized into functional groups and sub-groups, the members of each such group being intimately united together so that the excitement of any member of the group is shared by all the rest; such a group is the physiological counterpart of **a mental system**, or better, is the mental system in its physiological aspect (p. 606).

That an object is familiar to us means that, by successive presentations of its various aspects, the group of neurones directly excited by the presentation of any one aspect, has become intimately functionally related with the groups directly excited by the presentations of other aspects of the object. When we attend to any one aspect or feature we are apperceiving that feature, that is, we are relating it to other features, and this means in turn that the group of neurones directly excited by it is being brought into functional relation with those groups which have been previously organized by a similar process into the mental system, or complex group of neurones, corresponding to the total object.

The physiological process constantly accompanying or underlying the focal consciousness of states of mental activity is then a process by which one group of neurones is brought into more intimate functional relation with another, generally a more complex, group of neurones. Now so far as we can at all conjecture, such an association of two groups of neurones means a change at the **synapses** which constitute the physiological boundary and points of contact between the neurones of the one group and those of the other (i.e., physiological contact, not necessarily physical contact); a change of such a nature that thereafter any excitation can spread more readily across the synapses from one group to the other.

Such a change, such a diminution of the resistance at synapses, seems to be the normal result of the passage of the excitation process across them. [In a footnote on page 607, he says that: Of this we have fairly direct evidence in the shortening of reaction times with repetition; but beyond this, it is no exaggeration to say that without this assumption it is impossible to begin to attempt to correlate psychic and neural functions or to form the vaguest conception of the physiological change underlying association and constituting the **physical basis of memory**.

Focal consciousness seems, then, to have as a constant physiological accompaniment the process by which the excitement of one group of neurones overcomes the resistance of a set of synapses and spreads across them to another group; and as these synapses become organized, i.e., as their resistance becomes reduced more and more by repetition of the discharges across them, the consciousness accompanying the process becomes less and less clear and less intense until, when the synapses have become fully organized (i.e., have their resistance reduced to a minimum), it is of a very obscure nature, forming at most only a part of what William James (in his *Principles of Psychology*) has called “the fringe of thought”; for the two groups of neurones have then become one functional group, one mental system, and a presentation, that formerly directly excited the one group only, is now implicitly apprehended and automatically reacted to, the excitation-process set up by it spreads through the whole mental system without having to overcome the resistance of unorganized synapses, and without arousing focal consciousness (McDougall, 1901a, pp. 607, 608).

McDougall (1901a) then proposed that changes in synaptic resistance form the basis of associative memory:

Memory or other evidence of retention] means the persistence of some physical change in the conduction-paths. And the persistent change, which is the physical basis of memory or retention can only be conceived as consisting in such a union of the units composing a conduction-path that they shall in future tend to be simultaneously active, and this must mean an increased intimacy of connection between neurones, the result of **organization of the synapses** through which they act upon one another. Similarly, the physical basis of that form of retention known as the association of two ideas by contiguity in time can only be conceived as an organization of, a diminution in resistance of, the synapses through which the two conduction-paths, whose activity is the physical basis of the two ideas, can act upon one another. Such organization of synapses is, as we have seen reason to believe, the result of the passage of the excitation process across them, which passage across unorganized synapses is then the only physiological process known to be constantly accompanied by consciousness, and therefore in all probability constitutes or immediately determines the psychophysical process (McDougall, 1901a, p. 609).

In the concluding section of his paper, McDougall (1901a) reiterated his theory that the synapse, rather than the neuron, should be considered as the fundamental unit of neural activity, and suggested that a chemical substance, which he called “**neurin**” was released at the synapse to communicate between the pre- and post-synaptic neurons. Surely this must be the first chemical hypothesis of neural transmission underlying mental processes!

There is, then, a considerable mass of evidence, histological, physiological and psychological, pointing to the **synapses**, more especially those of the cerebral cortex, as the seats of the psycho-physical processes, while, so far as I can discover, there is no reason of any kind whatever to suppose that the cell-bodies are the seats of those processes, as has been so commonly assumed (pp. 609–610).

In place of regarding each neurone as having a certain threshold common to all its dendrites, each pair of fibrils that meet in a synapsis will be regarded as separated by an inter-cellular substance whose degree of resistance or threshold has been determined by the history of that synapsis in the individual and the species, and our conception of the mechanism that determines the course of any excitation will then be refined in proportion as synapses are more numerous than neurones.

The idea of a certain charging of neurones has been entertained by authors of high authority, but I have not been able to discover that anything has been said as to the nature of that something with which the neurones are supposed to be charged, although an effective working conception of its nature must be of the highest importance if there be any truth in this view. I think that for the present it may be best conceived as a fluid, and I propose that this fluid shall be called “**neurin**.” In a footnote on page 614, he says: “I am indebted for the suggestion of this word, to my friend G. C. Moore Smith” (about whom see Wilson, 1944). It might of course be called the nervous fluid, or nervous energy, or “animal-spirits,” or a very subtle ether, but the name I suggest is preferable because it implies nothing beyond the fact that the thing named has to do with nerves (McDougall, 1901a, p. 614).

Here then, is the first theory that a putative neurotransmitter, “neurin” transmits energy from one neuron to another at the nerve junction or synapse. McDougall is uncertain of his neurin concept, but he develops a theory of chemical transmitters in the nervous system, long before such substances were identified by Sir Henry Dale and Otto Loewi (see Valenstein, 2002). He said that:

I have little doubt that at some future date it will be regarded as some form of motion of some species of ether, or of some still more remote hypothetical medium. But just as the fluid-theory of heat, the two-fluid-theory of electricity and the corpuscular theory of light furnished probably the most useful working conceptions for the sciences of heat, electricity and light at certain stages of their development, so **neurin** may, I think, be most usefully conceived as a fluid in the present state of neurology, and I think it would be unwise to attempt to regard it as a variety of any one of the forms of energy known outside the animal body, although it is easy to discover points of resemblance to both electricity and magnetism (pp. 614, 615).

I will therefore sketch in rough outline a scheme of the part that the fluid **neurin** seems to play in the workings of the nervous system. In virtue of its normal vital activity every neurone continually produces neurin in small quantity; and the neurones connected with the sense-organs and surface of the body generally are almost perpetually played upon by feeble stimuli that excite them to the production of neurin in rather larger quantities. Neurin tends always to flow from a place of high potential to places of lower potential. The neurin so produced therefore tends continually to flow from afferent to efferent neurones, in virtue of the higher potential of the sensory neurones and of the valve like nature of the synapses, passing across the synapses by a sort of leakage, and so escaping by the efferent nerves into the muscles as a continuous gentle stream, maintaining that state of continued gentle contraction of the muscles which we call their tone (In a footnote on page 615 he says that: In this connection it should be remembered that in spite of the oft repeated statement to the contrary effect, a continuous current of constant strength is capable of bringing about a continued contraction when thrown into a muscle-nerve preparation under suitable conditions. It is possible that the slow leakage across synapses is the basis of that obscure affection of consciousness which has been called the “general sensibility” and forms the groundwork of the psychological self) (McDougall, 1901a, pp. 615, 616).

In his little book entitled *Physiological Psychology*, McDougall (1905) examined the neural theories of memory and reiterated his theory that memory did not occur in the nerve cells themselves, but in the connections between nerve cells. In discussing “Neural association” (pp. 124–139) he reviewed the “law of neural habit,” about which James (1890, in his chapter on Association, p. 566) said that “all the materials of our thought are due to the way in which one elementary process of the cerebral hemispheres tends to excite whatever other elementary process it may have excited at some former time.” McDougall (1905) went on to say that “there is another elementary causal law of association at least equally important with the law of neural habit, without the operation of which the latter would never have an opportunity to play its part in forming neural associations.” (p. 125). He then stated a precursor to the Hebb synapse theory:

This law may be stated thus: When the excitement of one neural system **a** is immediately followed by the excitement of another system **b**, the free nervous energy of the former system a tends to discharge itself by some path into the other **system b** (McDougall, 1905, pp. 125, 126).

In essence, this is a reformulation of William James’s “law of neural association through temporal contiguity”: “When two elementary brain-processes have been active together or in immediate succession, one of them on recurring tends to propagate its excitement into the other” (McDougall, 1905, p. 128). McDougall then outlines James’s theory for a neural basis for what we know as the conditioned avoidance response or Pavlovian conditioned reflex and then gives a critique of this theory in terms of his drainage theory of inhibition (McDougall, 1905, pp. 142–144).

### An Aside on the Early Names for Chemical Neurotransmitters

In his paper on the physiological basis for hypnosis, McDougall (1908c, p. 247) noted that Oscar Vogt (1870–1959) and August Forel (1848–1931) had used the term “Neurokyme” for the hypothetical chemical neurotransmitter at synapses and he decided to use this term rather than his term “neurin.” However, an editorial in the British Medical Journal **(10 Oct 1908)**, criticized McDougall’s attempt to provide a physiological theory of hypnosis based on the release of neurokyme, and this led to a series of letters on what to name the “nervous energy” released at a synapse. In a commentary in the British Medical Journal, White (1908) noted that he had coined the term “neurorrheuma” in 1886 for “the form of energy that is transmitted along a nerve.” This led Guthrie (1908) to provide a history of the terms used for “nervous energy” from the time of Hippocrates, and to decry the new names such as “Neurokyme” for something that is “beyond the reach of our capacities.”

However, all of these commentaries seemed to have ignored the discoveries of the chemist Beyer (1866, 1867) who determined the structure of choline and acetylcholine. When he added an acetyl group to choline, he called it “**Acetylneurin**.” Mott and Halliburton (1899) published an extensive study of the physiological actions of choline and neurine on the heart rate and blood pressure. They showed that **neurine** had very different properties than choline, and compared the effects of neurine with those of muscarine and nicotine. When McDougall (1901a) coined the term “neurin,” it appears that he did not know about the work of Beyer and Mott on “neurine.” Mott (1904, p. 1,556) was critical of “that hypothetical substance which McDougall has unfortunately called neurin” and in a footnote in his review of Sherrington’s *Integrative Action of the Nervous System*, Mott (1907, p. 570) explained that the term “neurin” is unfortunate because “this term is already applied to a toxic substance which may under certain conditions arise from nerve degeneration.” Thus, Mott felt that neurin was a neurotoxin, and when acetylcholine was first isolated, it was as an ergotoxine (Dale, 1914; Ewins, 1914) and in the conception of Langley and others, nicotine, curare and other drugs functioned as neurotoxins, not chemical signals. Thus, the term neurin seemed unfortunate but maybe McDougall knew something that Mott did not! Of course, neurine or acetylneurin was the substance called acetylcholine by Sir Henry Dale in 1914, and Vagusstoff by Otto Loewi in 1921. Of all the different names, acetylcholine stuck and so we know it today as the first chemical neurotransmitter that was identified (Tansey, 1991; Zigmond, 1999; Todman, 2008; López-Muñoz and Alamo, 2009; Sourkes, 2009).

### McDougall’s Theory of Memory Consolidation

McDougall (1901a) used his synaptic theory to explain the reflex arc, summation of stimulation and facilitation. He proposed that when sensory nerves were stimulated, they released “larger quantities of **neurin** per unit of time” and that this occurred in each neuron in a reflex arc, “from the sense-organ to any motor neurone,” “so that in a small fraction of a second the increase in the release of neurin will rise to the level of the threshold of the synapsis”:

A sudden discharge of **neurin** then takes place across the synapsis from **a to b**, and its sudden arrival in **b** acts upon it as a stimulus to the rapid production of more neurin, so that **b** then has a double charge of neurin and therefore rapidly discharges into **c**; and so the process of discharge and stimulation of afferent into efferent neurone is repeated, until the last of the chain discharges itself into the muscle and brings about contraction (McDougall, 1901a, p. 616).

McDougall then considered the findings of Sherrington and others on the summation of stimuli, on facilitation, and on the continuation of nervous activity after the stimulus ends, which Hebb (1949) called “reverberating circuits” after Lorente de No, but it seems that McDougall proposed this much earlier. About summation and facilitation, he said:

Stimuli far too weak to singly evoke reflex action, are easily able to do so if repeated (Sherrington, 1900. “**The Spinal Cord**,” **Schafer’s** “**Text-book**,” **pp**. **828**, **829**). Each stimulus causes the generation of a quantity of neurin in the sensory neurone too small to raise its potential to the synapsis-threshold, but, when the stimuli are repeated at such a rate that each fresh quantity is formed before any considerable fraction of the preceding charge has leaked away, the charges accumulate until the potential reaches the synapsis-threshold.

Exner (1894, pp. 131, 158) has shown that if a spot of skin and a spot on the motor cortex be found, stimulation of which leads to contraction of the same muscle in both cases, then weak stimuli applied to these two spots re-enforce one another whether they be separately sub-minimal or not, and whether they be simultaneous or successive at any interval less than 3 s. This is the typical instance of what Exner has called “Bahnung,” a word translated by Sherrington as “**facilitation**.”

The explanation in terms of **neurin** would seem to be entirely similar; in the case described the two conduction-paths, that from the spot on the skin and that from the cortex, converge at some point in their course to become one, and in this part, whose neurones are common to both paths, accumulation of charges of neurin takes place, whether through simultaneous charging from both directions or through **summation** of successive charges (McDougall, 1901a, pp. 618, 619).

McDougall (1901a) then applied his synaptic theory to the findings of Müller and Pilzecker (1900) on the “Perseverations-tendenz” or persistence of nervous activity following the learning of nonsense syllables, which was proposed to form the basis of the “fixity of the associations between the syllables.” As pointed out by Lechner et al. (1999), this paper by Müller and Pilzecker (1900) was the first description of memory consolidation. McDougall describes how his neurin theory of synaptic activity could account for this “Perseverations-tendenz” or memory consolidation:

The phenomena of facilitation and the summation of stimuli afford evidence of the persistence in neurones of a charge of neurin for some seconds, and there is other evidence pointing to the persistence of a residual charge after the discharge of neurones. In the case of the cord this is indicated by experiments which prove that the production of a reflex is rendered easier by an immediately preceding activity of the conduction-path concerned (Sherrington, 1900. “**The Spinal Cord**,” **in Schaefer’s** “**Text- book**,” p. 842).

Similar evidence in the case of neurones of the brain is afforded by certain psychological observations. In an important paper, **Müller and Pilzecker** have shown that an image (they dealt chiefly with nonsense-syllables) that has occupied consciousness tends to rise again to consciousness spontaneously, as it were, i.e., without any assignable reproducing association, a tendency to which they give the name “Perseverations-tendenz.” And they have further shown that during a similar period, following the succession of two images in consciousness, the association between those two images continues to increase in strength.

My suggestion is, that there remains in the group of central neurones concerned in the production of an image **a residual charge of neurin**, so that their potential remains not far below the discharging point, and a very slight stimulus will cause their discharge, and so the revival of the image in consciousness; and that the gradual escape of this residual charge, by leakage across synapses, is the cause of the continued diminution of resistance of the synapses, i.e., of increasing fixity of the association.

The same authors show that the time needed for the reproduction of an image by an associated image is much shorter immediately after the image has passed from consciousness than a few minutes later, while the certainty of the association is not correspondingly greater. This result also points to the persistence of a residual charge identical with that to which, as I suggest, the “Perseverations-tendenz is due.

Of these various cases that are so readily described in terms of neurin, those which indicate the persistence of a residual charge seem to me the most important, for they show that the activity of the neurones cannot be regarded as a mere propagation and augmentation of an impulse, as is commonly assumed to be the case in peripheral nerve-fibers, or as a wave movement of some unknown medium that sweeps through them, as Richet suggests; but that we must assume that there is generated in the neurones something comparable to a charge of heat or of statical electricity that can be stored up for a time and can leak slowly away (McDougall, 1901a, pp. 620, 621).

McDougall then brings up the question as to whether a memory-image is identical to a sensation or whether they differ in some way. What he proposed is very similar to the concept of a reverberating circuit. He says:

Let the cortical conduction-path concerned in the production of the sensation be schematically represented as simply as possible by a chain of three neurones, **a**, **b**, **and c**, afferent, central and efferent, respectively. Then, on stimulation of the sense-organ, the excitation spreads through the lower afferent neurones up to a and through the chain of **cortical neurones**, **a**, **b**, **c**, and so out to some motor center; and the discharges across **the synapses a–b and b–c** constitute the psycho- physical processes that determine the sensation. Now when by association the idea of the same object is reproduced as a memory-image, the **neurone b** is excited not from **a**, but through some neurone of its own level, and it discharges through **synapsis b–c** as before. We may then suppose that the psycho-physical processes at synapses **a–b and b–c** are identical in quality and that the peculiar and undefinable quality that distinguishes the sensation from the idea, the presentation from the representation, is due simply to the duplication of the psycho-physical process; or more probably that, while **the processes at a–b and b–c** are essentially similar in quality, they yet differ in some respect, probably in that the **discharge of neurin at a–b** takes place at a higher potential than the discharge at **b–c**. In this way we may understand why it is that, while there are so many urgent grounds for regarding the seat of the sensation and of the idea as identical, yet on other grounds such a view is shown to be untenable; there is partial identity of “locus,” the “locus” of the idea is a part only of the “locus” of the sensation (McDougall, 1901a, pp. 622, 623).

Finally, McDougall (1901a) proposed a mechanism for synaptic changes in neural systems to form memories. He first distinguished between the synapse and the nerve cell body, and then proposed that synaptic changes were the mechanism for associative memory.

What exactly is the nature of the process of discharge of neurin across the synapses and what is the part played by the inter-cellular substance can hardly be so much as conjectured, but we must assume that each discharge constitutes, or brings about in the inter-cellular substance, the delicate and specifically differentiated psycho- physical process. And we must further assume that each such discharge leaves the substance so changed that its resistance is permanently lowered.

This permanent lowering of the threshold of the synapsis may be conceived to consist in any one of various changes, it may be that the thickness of the layer of inter- cellular substance between the terminals of the nerve-fibrils is diminished, or that its cross-section is increased, it may consist in some state of strain analogous to the strain of a dielectric substance remaining after discharge from a condenser, or in some molecular rearrangement, or more probably in some change of whose nature we cannot at the present time form any conception. This change, which seems to be **the physical basis of memory** and association, this organization of the synapses, which corresponds to what Exner calls “Ausfahren” or “Ausschleifung der Bahnen,” must be carefully distinguished from facilitation (Bahnung), which is a temporary condition due to the presence of a charge of neurin in the neurones. Exner himself observes the distinction, but by other writers the two things have been confused.

My proposition, that the synapses are the seats of the psycho-physical processes, involves the corollary that the state of consciousness of an individual at any moment is determined by several, and, in any but the simplest states, by a very large number of psycho-physical processes occurring simultaneously in different parts of the cerebral cortex, the total state of consciousness being the resultant of purely psychical fusion of the several affections of consciousness determined at the several synapses. **I am aware that this will not be readily accepted by many minds** that have not realized that psychical fusion is a conception absolutely necessary for any but the most superficial consideration of the relation of physical and psychical processes (McDougall, 1901a, pp. 625, 626).

### McDougall’s Application of His Synaptic Theory to a Range of Psychological Processes

In a series of four papers, McDougall (1902, 1903b,1903c, 1906), expanded on his ideas of the synapse and of “neurin” in the attention-process. In the first of these papers McDougall (1902), reviewed his theory of synaptic action and the release of “neurin” and then examined two theories on the location of memory: in the nerve cell itself, or in the synaptic connections between nerve cells. The engram theory of Semon (1921) can be considered as localizing memories within cells or groups of cells, while McDougall focuses on synaptic changes, which is Hebb’s theory. McDougall (1902, pp. 350, 351) ends this paper with his “note on neurin,” in which he replies to the critics of his proposal of a chemical neurotransmitter at the synapse.

In his second paper on the physiological factors underlying attention, McDougall (1903b) used his synaptic theory to examine the physiological basis of sleep, dreaming, and waking with respect to attention and consciousness and proposed that neurin provided the energy to keep neurons active during the waking state. His theory was that, due to fatigue, the level of neurin in the synapse is decreased, which results in sleep, and when the level of neurin is increased during sleep, or sensory stimuli activate the sensory nerves to release neurin, this increase in neurin activates the nervous system and the person wakes up. In his third paper on the physiological factors of the attention-process McDougall (1903c) examined muscular activity as a factor in the attention process by focusing on eye movements during visual attention. In the fourth and final paper in this series McDougall (1906) outlined his thoughts on “the existence of organized neural dispositions, corresponding on the neural side to the mental dispositions or mental or apperceptive systems” underlying sensory perception; i.e., the role of the brain in sensory perception. He examined the role of fatigue in attention and reviewed his previous ideas on the drainage theory of inhibition. On page 349 he stated that: “Physiologists are not agreed as to the nature of the processes of inhibition in the central nervous system.” This seems to have been an understatement.

McDougall (1908b, c) examined the physiological basis of hypnosis through the application of “the theory of cerebral dissociation” and then, in his final paper in this series, McDougall (1909) proposed a theory of fatigue, which involved the level of resistance at synapses. Although he did not mention neurin or neurokyme here (except in a footnote on page 264) he did refer to “nervous energy” and his theory of fatigue relied on the decrease of such nervous energy at the synapse. He stated that:

If we assume that the synapses are the seats of the resistances, and that the process of transmission of the impulse across the synapse is one that results in a temporary raising of the resistance of the synapse, then we have a conception which enables us to account satisfactorily for many manifestations of fatigue (McDougall, 1909, p. 260).

It is worth noting that Sherrington (as Chairman) and McDougall (as Secretary) worked together on the committee of the British Association for the Advancement of Science that was set up to study mental and muscular fatigue and McDougall had developed an apparatus to test mental fatigue, the Rivers-McDougall fatigue apparatus (Sherrington et al., 1911, 1912).

### McDougall’s Theory of Inhibition by Drainage

McDougall (1901a, pp. 623, 624) briefly proposed a theory of inhibition based on the blockage of the release of neurin at the synapse. This was important because, in a nervous system where communication between cells was only thought to be through electrical synapses, nobody could explain inhibition. In his next paper, McDougall (1903a) focused on the nature of inhibition in the nervous system and described his hypothesis of “inhibition by drainage.” He debated two possible physiological mechanisms of inhibition: (1) Inhibition due to “a cutting off or diminution within the nervous system of the excitatory impulses issuing to the muscles along the motor nerves,” and (2) “special inhibitory nerve fibers or the passage of inhibitory impulses along the motor nerves” (McDougall, 1903b, p. 161). Here McDougall backed the wrong horse. He was not in favor of the existence of special inhibitory neurons, and said that “If we assume that inhibition is brought about through special nerve-fibers we shall then have to suppose that all parts are interconnected by a duplicate set of paths, the excitatory and the inhibitory, that, in fact, almost the whole central nervous system exists in duplicate. Such an anatomical basis for the inhibitory processes would be a very clumsy and complicated affair, and not only is its existence, therefore, extremely improbable, but it is impossible that, if it existed, we should not have obtained some direct evidence of the fact” (McDougall, 1903b, p. 163). McDougall was not far wrong here. GABA was not identified as an inhibitory neurotransmitter in the brain until 1950 the 1950’s (Harris-Warrick, 2005; Bowery and Smart, 2006; Spiering, 2018). Since neurotransmitters had not yet been discovered, and McDougall’s neurin hypothesis focused only on excitatory synapses, he concluded that inhibition was due to lack of excitation, and said that:

We find ourselves, therefore, driven to adopt the view that inhibition in the greater part of the nervous system, all the part concerned in the control of the skeletal musculature, consists simply in the cutting off from the tract inhibited of the excitatory impulses by which alone its activity can be maintained. When we seek an hypothesis as to the mechanism by which this cutting off of excitatory influences may be effected we find that clear and unmistakable indications are afforded by certain psychological considerations (McDougall, 1903a, p. 167).

The “drainage theory” of inhibition proposed by McDougall (1903b, p. 167) was based on the experiments of Sherrington but derived from William James. He stated that:

several physiological-psychologists foreshadow more or less vaguely the hypothesis that inhibition is due to competition among nerve-paths for the living energy present at the moment in the nervous system. The most definite presentation of this conception that I have read is contained in those most interesting pages which conclude. James’ chapter on the Will (“*Principles of Psychology*,” vol. ii., p. 579, et seq.) in which he writes of inhibition **through drainage** of one cell by another.

The basis of McDougall’s belief in the drainage theory of inhibition was that energy was distributed through the synapses via “neurin.” He noted that there must be a distinction between potential energy and living energy within the nervous system, and said that:

It is because I feel so strongly the need for a clear conception of the living energy as distinguished from the chemical potential energy of the nervous system that I have suggested the name “**neurin**” in place of the cumbrous phrase “living nervous energy,” believing that (1) to give a distinctive name to any conception is an important step toward rendering it clear and definite; (2) that the living energy flows in currents from part to part, and in so doing follows the paths of least resistance; (3) the fact that the energy of the nervous system as a whole is more or less at the disposal of every part; (4) that **inhibition** means a switching off of the current of energy or **neurin**, and that a movement of attention means the switching off of the current from one path of forward-conduction in the higher levels of the brain and the turning of it into some other similar path which, through a complex constellation of influences, has become at that moment the path of least resistance in those higher levels (McDougall, 1903b, p. 171).

He also used his neurin theory to explain different types of inhibition, including the reciprocal inhibition studied by Sherrington:

The relation of **reciprocal inhibition** obtaining between all the different organized neural systems, constituting paths of forward-conduction in the higher brain- levels, is due, I suggest, to the fact that they all drain one common store of **neurin** contained and constantly generated in the interconnected mass of afferent neurones, and seeking constantly to escape by the paths of least resistance into motor neurones, and so into the muscles. The higher-level paths are brought into activity only when the store of neurin attains a certain potential or head of pressure, which degree of pressure is an essential condition of attentive consciousness. And only one of these higher-level paths can be active at any one moment, because any one of them is capable of carrying off the whole surplus of **neurin;** when, then, any combination of causes reduces the resistance of any other path to a lower point than that of the path active at any moment, the current shifts from one to the other, just as the opening of a new and shorter channel for a river causes the stream to flow wholly in the new channel, leaving in the old river bed only stagnant pools of water (McDougall, 1903b, p. 172).

McDougall (1906, p. 350) summarized his drainage theory of inhibition as follows:

The excitement of any sensori-motor arc diminishes the resistance that it offers to the onward passage toward its efferent neurones of the current of free nervous energy or **neurin**, and its resistance is the more diminished the greater the intensity of its excitement. If then two or more such arcs are connected together in their central parts, that one which is the most intensely excited will become for the time being the path of lowest resistance for the escape of neurin to the motor neurones, and will therefore tend to drain to itself and to discharge by way of its motor neurones the energy liberated in all the others.

## Did Anyone Pay Attention to Mcdougall’s Synaptic Theory?

Although much has been written about McDougall’s influence on Psychology (Burt, 1939, 1955; Greenwood and Smith, 1940), most of the emphasis was on his contributions to social psychology (Heidbreder, 1939) and abnormal psychology (Holsopple, 1939) and these say virtually nothing about his early work on the physiological basis of mental events. Adams (1939, p. 2) pointed out that “McDougall is not so well known to the public through his contributions to scientific psychology as he is through the instinct theory which he has elaborated” but says nothing of these scientific contributions. McDougall’s early work on visual perception was largely ignored (see Wu, 2012) as was his work on the synaptic theory of cognitive function, except for brief mentions by Pickworth (1938) and Langfeld (1940). Innis (2003) summarized McDougall’s work on the physiological basis of mental processes, pointed out his stress on the importance of the synapse in psychophysical activity, and mentioned his theory that “every psychical state corresponds to the flow of neurin through a certain set of neurons,” but she did not elaborate on his theories.

However, McDougall’s synaptic theory was not completely ignored. In his 1904 Silliman lectures at Yale University, published as *The Integrative Action of the Nervou*s System, Sherrington (1906b) made numerous references to McDougall’s work and cited six of McDougall’s papers. In particular, Sherrington (1906b, pp. 202–204) discussed McDougall’s theory of reciprocal inhibition by drainage through the release of neurin at synapses and reprinted (McDougall, 1903a, p. 175) figure describing this theory (Sherrington, 1906b, Figure 56, p. 202). Sherrington and McDougall had a long association, from the time that McDougall worked with Sherrington on muscle activity at St Thomas’ Hospital between 1894 and 1898 to their teaching in the physiology program and UCL in 1902 and later on the committee on fatigue between 1906 and 1911. Quick (2017, pp. 440–444) discusses the relationship between Sherrington and McDougall. Sherrington (1906b, p. 204) did, however, find two difficulties with McDougall’s theory: first, it did not explain how *a* comes to inhibit *b*, and second, it dissociated inhibition in the CNS and neuromuscular system from inhibition in other physiological systems such as the heart, blood vessels, and viscera.

Others who attended to McDougall’s physiological theories, also focused on his hypothesis of inhibition by drainage. Dodge (1925, 1926) reviewed six theories of inhibition, including the drainage theory of McDougall, and gave a critical evaluation of the theory and the data proposed to support it. He then provided data from more recent experiments and concluded that, the theoretical difficulties and experimental data presented “render the drainage theory of inhibition highly improbable” (Dodge, 1926, p. 122). Lashley (1924, p. 369) rejected the idea that synaptic change could underly learning and repeated his critique of synaptic resistance theory in his book on *Brain Mechanisms in Intelligence*, where he said that “it is not clear that the synapse is either essential or important for learning” (Lashley, 1929, pp. 125–127). Since Lashley did not believe that synaptic plasticity was involved in learning, it stands to reason that he would reject a synaptic theory of inhibition.

Lashley (1929, p. 164) dismissed drainage theory, saying that “Of the earlier theories, and by far the most elaborate one, on the nature of nervous energy is that of McDougall (1903a: The nature of inhibitory processes). He postulated a special kind of energy (neurin) associated with the activity of the nervous system, capable of storage in reserve, of drainage through open synapses, and of diversion into any one of a number of channels to facilitate various activities. The theory has much to recommend it as an interpretation of psychological data but seems flatly contradicted by more recent data upon the nature of the propagated disturbance in the nerve fiber.” Lashley (1934, p. 491) stated that “The theory of drainage is opposed by all of the more recent data upon the nature of the propagated disturbance in nerves.” In his textbook on *Physiological Psychology*, Morgan (1943, p. 523) also dismissed McDougall’s drainage theory, but was more favorable to the theory that synaptic resistance may play a factor in learning (pp. 520, 521) not mentioning that this was the central feature of McDougall (1901a) theory! McDougall (1926b) wrote a rebuttal to Dodge’s criticism of his drainage theory and in discussing how his drainage theory could explain Pavlov’s work on the problem of inhibition, McDougall (1929) also gave rebuttals to the critiques of Sherrington (1906b) and Lashley (1924, 1929).

On the other hand, in his history of psychology Flugel (1933/1964, p. 228) states that McDougall’s synaptic theory of consciousness, including his theory of inhibition by drainage was “the most successful neurological theory that has ever been proposed”; an opinion echoed by Cyril Burt (1939, 1955), a former student of McDougall. As discussed by Brown (2020), Hebb had no concept of inhibition in his cell assembly hypothesis, although he did consider the problem of inhibition in early drafts of his theory. Hebb (1980, p. 101) said that “the existence of a neural inhibition was not definitely known in 1949 and was not incorporated in the theory though it would have helped. Milner (1957) showed how to incorporate it.” Conclusive evidence for inhibitory neurons was not available until the concept of chemical neurotransmitters was accepted and inhibitory synapses were identified in 1954 (Eccles, 1990).

## Other Physiological Theories of Learning and Memory

As discussed by Brown (2020), there were a number of other synaptic theories of learning and memory in the early 1940’s which were outlined by **Hilgard and Marquis (1940**, **pp**. **326–335**) and Morgan (1943, pp. 520–525). These theories included “Neurobiotaxis,” the formation of new synapses; “Synaptic resistance,” the reduction of synaptic resistance during learning; “Fiber conductance,” the theory that repeated passage of an impulse along an axon increased its conductivity; “Reverberation,” the activation of reverberatory nerve circuits; and “Resonance,” the idea that neurons that are out of tune, become more in tune with each other during learning. There were also the Gestalt memory trace theories (Koffka, 1935; Köhler, 1947) and later, Konorski’s (1948) theory of synaptic plasticity underlying learning (Bijoch et al., 2020).

Semon’s engram theory was a form of “organic memory” which associated learning and memory in the individual with genetic memory and the inheritance of acquired characteristics (see Logan, 2015). Semon wrote three books, two of which, *The Mneme* (1921) and *Mnemic Psychology* (Semon, 1923) are often cited as the basis for the engram theory of memory, but the third, “*The Problem of the Inheritance of Acquired Characteristics*” (Semon, 1912) has been completed neglected. To Semon, the engram had an evolutionary function and was “the key linking cognitive memory with heredity” (Logan, 2015, p. 418). The philosophical discussion of the modern use of the engram theory to study the neural basis of memory using optogenetics is discussed by Robins (2018).

Holt (1931, p. 30) used the terms “neural engram” and “neurogram” and also presented an early version of the synaptic theory of learning, which sounds very much like those of James and of McDougall. Holt (1931, p. 34) stated that “Engrams or special pathways for nerve impulses, are inscribed in this diffuse network (of neurons) by a lowering of the resistance of some, as compared with other, synaptic junctions. Though the electrochemistry of this process is imperfectly understood, the fact is sufficiently attested that every passage of a nervous impulse across the junctional tissue between two neurons (the synapse) lowers the resistance of that tissue to the passage of all subsequent nervous impulses” (see Brown, 2020).

### Neuroscientists Before Their Time

Gross (2009) discussed the ideas of Emanuel Swedenborg (1688–1772) on the functions of the cerebral cortex, the theory of internal bodily homeostatsis of Claude Bernard (1813–1878), and the theory of adult neurogenesis of Joseph Altman (1925–2016), all of which were “ideas before their time,” “ignored by their contemporaries but accepted as major insights decades or even centuries later.” Finger (1994) book on the *Origins of Neuroscience* abounds with other theories of brain function that were not accepted when first presented. However, new discoveries are often controversial and result in debates such as those between Galvani and Volta on the theory of animal electricity (Piccolino, 1998) and between Golgi and Cajal on the neuron doctrine (Guillery, 2005, 2007). The debate on whether or not neural signaling involved chemical neurotransmitters continued for decades (Valenstein, 2002). Studying the history of neuroscience helps to understand how new discoveries became accepted or rejected (Brown, 2019). The theories of Semon (1921) which included the “engram” were also ignored for many years (Schacter et al., 1978), but “Semon’s concept of the engram was enmeshed in a complex theory of heredity, instinct, learning and evolution, and behavioral psychologists who studied the neural basis of memory slowly dissociated the use of the term “engram” from Semon’s overall theory and used it to mean the neural location of a memory” (Brown, 2019, p. 22). It is clear from what we have written above that McDougall’s synaptic theory of cognitive function also fits into the category of ideas that were ahead of their time.

## Conclusion. What Have We Learned? There Is Nothing New Under the Sun

In this manuscript we have given an overview of the theories of the neural basis of learning and memory before Hebb and described the synaptic theory of McDougall, which appears to have been an idea ahead of its time and ignored when it was first presented. Before any physiology-based neural theories of mental functions could be developed, brain researchers had to embrace cell theory, the neuron doctrine and the concept of the synaptic junction between neurons. McDougall had extensive experience in the physiological psychology of his day and was trying to “update” the theories of James by adding “modern” neurophysiological ideas: the synapse and the chemical transmitter “neurin,” as well as a physiological basis for the concepts of summation, facilitation and inhibition. In many ways, McDougall was trying to integrate the ideas of William James, Charles Sherrington, and Georg Elias Müller.

McDougall (1901a) seems to have been the first person to use the term “synapse” to explain the basis of the neural changes underlying experience. It appears that McDougall’s theories of neural function were ignored because they were too far ahead of their time. At Oxford, McDougall fell between the cracks of physiology and mental philosophy and was accepted by neither. Americans, particularly, ignored McDougall because, by the time he arrived in Harvard in 1920, he was best known for his book on social psychology in which he proposed that all human behavior, especially social behavior, was based on instincts, which was an anathema to American psychologists who were embracing behaviorism. McDougall believed in “purposive psychology” and in 1924 debated Watson on Behaviorism vs purposive psychology. He did research on Lamarckian inheritance, and was a confirmed believer in psychic phenomena, providing plenty of evidence for his statement that he was opposed to the popular or orthodox views in psychology, and as a consequence, his contributions were ignored. Heidbreder (1939, pp. 151, 152) says this very clearly:

For the plain fact is that to many of his fellow psychologists, McDougall stood outside the pale of scientific respectability. In America he was widely regarded as an anachronism and a menace; his name became almost synonymous with theories and practices regarded by most American psychologists as remnants of exploded but still dangerous superstitions—with animism, vitalism, and teleology; with nativism in the discredited form of Lamarckism; and with shady ventures into psychical research and extra-sensory perception. Perhaps more than any other one individual, McDougall became a symbol of what American psychology has most heartily set itself against.

Although Sherrington, Dodge, and Lashley critiqued McDougall’s theory of inhibition by drainage, they paid little attention to McDougall’s earlier papers. Even if they had, they would have rejected his synaptic resistance theories of neural function. However, McDougall made a number of proposals which were later demonstrated to be on the right track. These were (1) the importance of the synapse versus the cell body in neural plasticity, which is still contentious; (2) the release of a chemical substance to communicate between neurons at a synapse; (3) a theory of inhibition, which although erroneous, was consistent with his theory of synaptic function. (4) A mechanism for synaptic activity after the offset of a stimulus, thus an early proposal for Hebb’s reverberating circuits and (5) a mechanism for memory consolidation. In terms of the emerging engram theory, McDougall rejected the idea that memory resided inside a nerve cell, which is the theory implicit in the engram theory of Semon, while he embraced the theory of synaptic change, which was rejected by Lashley and embraced by Hebb. Still, it is puzzling why every biography of McDougall omitted his synaptic theories, except Burt, where they got short shrift. Even Boring (1950, pp. 465–476) says little about McDougall’s theories of physiological psychology. We hope that this manuscript will encourage a re-examination of the synaptic theory of McDougall in light of later advances in the neurobiology of behavior.

## Author Contributions

REB, TB, and JFG wrote the manuscript. REB edited and supervised the project. All authors contributed to the article and approved the submitted version.

## Conflict of Interest

The authors declare that the research was conducted in the absence of any commercial or financial relationships that could be construed as a potential conflict of interest.

## Publisher’s Note

All claims expressed in this article are solely those of the authors and do not necessarily represent those of their affiliated organizations, or those of the publisher, the editors and the reviewers. Any product that may be evaluated in this article, or claim that may be made by its manufacturer, is not guaranteed or endorsed by the publisher.

## References

[B1] Abraham W. C. Jones O. D. Glanzman D. L. (2019). Is plasticity of synapses the mechanism of long-term memory storage? *NPJ Sci. Learn.* 4:9. 10.1038/s41539-019-0048-y 31285847 PMC6606636

[B2] Adams D. K. (1939). William McDougall. *Psychol. Rev.* 46 1–8. 10.1037/h0056620

[B3] Anonymous (1902). The University of London. research and teaching in advanced physiology in London. *Br. Med. J.* 1 213–214. 10.1136/bmj.1.2143.213 20760021 PMC2511804

[B4] Basile P. (2017). “James Ward,” in *The Standford Encyclopedia of Philosophy*, ed. Zalta E. N. (Stanford, CA: The Metaphysics Research Lab, Stanford University).

[B5] Batool S. Raza H. Zaidi J. Riaz S. Hasan S. Syed N. I. (2019). Synapse formation: from cellular and molecular mechanisms to neurodevelopmental and neurodegenerative disorders. *J. Neurophysiol.* 121 1381–1397. 30759043 10.1152/jn.00833.2018

[B6] Bayliss W. M. Starling E. H. (1902). The mechanism of pancreatic secretion. *J. Physiol.* 28 325–353. 10.1113/jphysiol.1902.sp000920 16992627 PMC1540572

[B7] Bennett M. R. (1999). The early history of the synapse: from Plato to Sherrington. *Brain Res. Bull.* 50 95–118. 10.1016/s0361-9230(99)00094-5 10535329

[B8] Berlucchi G. Buchtel H. A. (2009). Neuronal plasticity: historical roots and evolution of meaning. *Exp. Brain Res.* 192 307–319. 10.1007/s00221-008-1611-6 19002678

[B9] Beyer A. (1866). Synthese des Neurins. *Annalen Chemie Pharmacie* 140 306–313. 10.1002/prac.18681050162

[B10] Beyer A. (1867). Üeber das Neurin. *Annalen Chemie Pharmacie* 142 322–326. 10.1002/jlac.18671420311

[B11] Bijoch L. Borczyk M. Czajkowski R. (2020). Bases of Jerzy Konorski’s theory of synaptic plasticity. *Eur. J. Neurosci.* 51 1857–1866. 10.1111/ejn.14532 31368131

[B12] Black S. E. (1981). Pseudopods and synapses: the amoeboid theories of neuronal mobility and the early formulation of the synapse concept, 1894-1900. *Bull. Hist. Med.* 55 34–58. 7011452

[B13] Bock O. (2013). Cajal, Golgi, Nansen, Schäfer and the neuron doctrine. *Endeavour* 37 228–234. 10.1016/j.endeavour.2013.06.006 23870749

[B14] Boring E. G. (1950). *A History of Experimental Psychology*, 2nd Edn. New York, NY: Appleton-Century-Crofts.

[B15] Bowery N. G. Smart T. G. (2006). GABA and glycine as neurotransmitters: a brief history. *Br. J. Pharmacol.* 147 S109–S119. 10.1038/sj.bjp.0706443 16402094 PMC1760744

[B16] Broca P. (1861). Nouvelle observation d’aphémie produite de la moitié postérieure des deuxième et troisième circonvolutions frontales gauches. *Bull. Soc. Anatomique* 36 398–407.

[B17] Brodmann K. (1909). *Vertgleichende Lokalisationslehre der Grosshirnrinde.* Leipzig: J.A. Barth.

[B18] Brown R. E. (2017). “Revisiting Hebb: the organization of behavior,” in *Brain and Behaviour*, eds Kolb B. Whishaw I. (Thousand Oaks, CA: SAGE Publications Ltd), 69–93. 10.4135/9781529715064.n7

[B19] Brown R. E. (2019). Why study the history of neuroscience? *Front. Behav. Neurosci.* 13:82. 10.3389/fnbeh.2019.00082 31191266 PMC6539194

[B20] Brown R. E. (2020). Donald O. Hebb and the organization of Behavior: 17?years in the writing. *Mol. Brain* 13:55. 10.1186/s13041-020-00567-8 32252813 PMC7137474

[B21] Brown R. E. Milner P. M. (2003). The legacy of Donald O. Hebb: more than the Hebb synapse. *Nat. Rev. Neurosci.* 4 1013–1019. 10.1038/nrn1257 14682362

[B22] Bruce D. (2001). Fifty years since Lashley’s in search of the engram: refutations and conjectures. *J. Hist. Neurosci.* 10 308–318. 10.1076/jhin.10.3.308.9086 11770197

[B23] Buchanan F. (1908). On the time taken in transmission of reflex impulses in the spinal cord of the frog. *Quart. J. Ex. Physiol.* 1 1–66.

[B24] Burnham W. H. (1888). Memory, historically and experimentally considered. I. an historical sketch of the older conceptions of memory. *Am. J. Psychol.* 2 39–90. 10.2307/1411406

[B25] Burnham W. H. (1889a). Memory, historically and experimentally considered. II. modern conceptions of memory. *Am. J. Psychol.* 2 225–270.

[B26] Burnham W. H. (1889b). Memory, historically and experimentally considered. IV. *Am. J. Psychol.* 2 568–622. 10.2307/1411858

[B27] Burt C. (1939). William McDougall: an appreciation. *Br. J. Educ. Psychol.* 9 1–7. 10.1111/j.2044-8279.1939.tb03189.x

[B28] Burt C. (1955). The permanent contributions of McDougall to psychology. *Br. J. Educ. Psychol.* 25 10–22. 10.1111/j.2044-8279.1955.tb01331.x

[B29] Cavero I. Guillon J.-M. Holzgrefe H. H. (2017). Reminiscing about Jan Evangelista Purkinje: a pioneer of modern experimental physiology. *Adv. Physiol. Educ.* 41 528–538. 10.1152/advan.00068.2017 29066603

[B30] Cimino G. (1999). Reticular theory versus neuron theory in the work of Camillo Golgi. *Physics* 36 431–472. 11640243

[B31] Cocquyt T. Zhou Z. Plomp J. Eijck L. V. (2021). Neutron tomography of Van Leeuwenhoek’s microscopes. *Sci. Adv.* 7:eabf2402. 10.1126/sciadv.abf2402 33990325 PMC8121416

[B32] Cooper S. J. (2005). Donald O. Hebb’s synapse and learning rule: a history and commentary. *Neurosci. Biobehav. Rev.* 28 851–874. 10.1016/j.neubiorev.2004.09.009 15642626

[B33] Dale H. H. (1914). The action of certain esters and ethers of choline, and their relation to muscarine. *J. Pharmacol. Exp. Ther.* 6 147–190.

[B34] De Carlos J. A. Borrell J. (2007). A historical reflection of the contributions of Cajal and Golgi to the foundations of neuroscience. *Brain Res. Rev.* 55 8–16. 10.1016/j.brainresrev.2007.03.010 17490748

[B35] De Carlos J. A. Molnár Z. (2020). Cajal’s interactions with Sherrington and the croonian lecture. *Anatom. Rec.* 303 1181–1188. 10.1002/ar.24189 31172626

[B36] de Castro F. (2019). Cajal and the Spanish neurological school: neuroscience would have been a different story without them. *Front. Cell. Neurosci.* 13:187. 10.3389/fncel.2019.00187 31178695 PMC6542961

[B37] Deiters V. S. Guillery R. W. (2013). Otto Friedrich Karl Deiters (1834-1863). *J. Comp. Neurol.* 521 1929–1953. 10.1002/cne.23316 23436306

[B38] Devan B. D. Berger K. McDonald R. J. (2018). The Emergent Engram: a historical legacy and contemporary discovery. *Front. Behav. Neurosci.* 12:168. 10.3389/fnbeh.2018.00168 30131682 PMC6090515

[B39] Dodge R. (1925). The hypothesis of inhibition by drainage. *Proc. Natl. Acad. Sci. U S A.* 11 689–691. 10.1073/pnas.11.11.689 16576928 PMC1086213

[B40] Dodge R. (1926). The problem of inhibition. *Psychol. Rev.* 33 1–12. 10.1037/h0072125

[B41] Eccles J. C. (1990). Developing concepts of the synapses. *J. Neurosci.* 10 3769–3781. 10.1523/JNEUROSCI.10-12-03769.1990 1980129 PMC6570050

[B42] Edgell B. (1947). The British psychological society. *Br. J. Psychol.* 37 113–132. 10.1111/j.2044-8295.1947.tb01127.x 20241968

[B43] Eichenbaum H. (2018). Barlow versus Hebb: when is it time to abandon the notion of feature detectors and adopt the cell assembly as the unit of cognition? *Neurosci. Lett.* 680 88–93. 10.1016/j.neulet.2017.04.006 28389238 PMC5628090

[B44] Elliott T. R. (1904). On the action of adrenalin. *J. Physiol.* 32 401–467. 10.1113/jphysiol.1905.sp001093 16992786 PMC1465728

[B45] Ewins A. J. (1914). Acetylcholine, a new active principle of ergot. *Biochem. J.* 8 44–49. 10.1042/bj0080044 16742288 PMC1276542

[B46] Exner S. (1894). *Entwurf zu einer Physiologischen Erklärung der Psychischen Erscheinungen.* F. Deuticke: Leipzig.

[B47] Ferrier D. (1876). *The Functions of the Brain*, 2nd Edn. London: Smith, Elder & Co.

[B48] Finger S. (1994). *Origins of Neuroscience: a History of Explorations into Brain Function.* New York, NY: Oxford University Press.

[B49] Flugel J. C. (1933/1964). *A Hundred Years of Psychology. (Revised by D. J. West).* London: University Paperbacks, Methuen. (Originally published in 1933 by Gerald Duckworth, London UK).

[B50] Foster M. Sherrington C. S. (1897). *A Text Book of Physiology: Part III. The Central Nervous System*, 7th Edn. London: Macmillan.

[B51] Franz S. I. (1912). New phrenology. *Science* 35 321–328. 10.1126/science.35.896.321 17774377

[B52] Freud S. (1895/1953). “Project for a scientific psychology,” in *Standard Edition of the Complete Works of Sigmund Freud*, ed. Strachey J. (London: Hogarth).

[B53] Fritsch G. Hitzig E. (1870). Über die elektrische Erregbarkeit des Großhirns. *Arch. Anatomie Physiol. Wissenschaftliche Med.* 37 300–332.

[B54] Gotch F. Horsley V. (1891). Croonian Lecture. On the mammalian nervous system, its functions, and their localisation determined by an electrical method. *Phil. Trans. Roy. Soc. Lond. B* 182 267–526.

[B55] Grant G. (2007). How the 1906 Nobel Prize in Physiology or Medicine was shared between Golgi and Cajal. *Brain Res. Rev.* 55 490–498. 10.1016/j.brainresrev.2006.11.004 17306375

[B56] Greenwood M. Smith M. (1940). William McDougall, Obituary notices. *Biog. Mem. Fell. Roy. Soc.* 3 39–62. 10.1098/rsbm.1940.0005

[B57] Gross C. G. (2009). Three before their time: neuroscientists whose ideas were ignored by their contemporaries. *Exp. Brain Res.* 192 321–334. 10.1007/s00221-008-1481-y 18641979

[B58] Guillery R. W. (2005). Observations of synaptic structures: origins of the neuron doctrine and its current status. *Phil. Trans. Roy. Soc. Lond. B, Biol. Sci.* 360 1281–1307. 10.1098/rstb.2003.1459 16147523 PMC1569502

[B59] Guillery R. W. (2007). Relating the neuron doctrine to the cell theory. should contemporary knowledge change our view of the neuron doctrine? *Brain Res. Rev.* 55 411–421. 10.1016/j.brainresrev.2007.01.005 17300841

[B60] Guthrie L. (1908). Neurorrheuma” or “Neurokyme. *Br. Med. J.* 2 1404–1405.

[B61] Harris-Warrick R. (2005). Synaptic chemistry in single neurons: GABA is identified as an inhibitory neurotransmitter. *J. Neurophysiol.* 93 3029–3031. 10.1152/classicessays.00026.2005 15911885

[B62] Hebb D. O. (1949). *The Organisation of Behaviour.* New York, NY: John Wiley & Sons.

[B63] Hebb D. O. (1972). *Textbook of Psychology.* Philadelphia, PA: Saunders.

[B64] Hebb D. O. (1980). *Essay on Mind.* Hillsdale NJ: Erlbaum.

[B65] Heidbreder E. (1939). William McDougall and Social Psychology. *J. Abnor. Soc. Psychol.* 34 150–160. 10.1037/h0056726

[B66] Hellman H. (2001). *Great Feuds in Medicine. Chapter 6. Golgi versus Ramon y Cajal.* New York, NY: John Wiley & Sons.

[B67] Hilgard E. R. Marquis D. G. (1940). *Conditioning and Learning.* New York, NY: Appleton-Century.

[B68] Holsopple J. Q. (1939). William McDougall and abnormal psychology. *J. Abnor. Soc. Psychol.* 34 161–165. 10.1037/h0058628

[B69] Holt E. B. (1931). *Animal Drive and the Learning Process, an Essay Toward Radical Empiricism.* New York, NY: Henry Holt and Company.

[B70] Hooke R. (1665). *Micrographia.* London: John Martyn & James Alleftry.

[B71] Hübener M. Bonhoeffer T. (2010). Searching for engrams. *Neuron* 67 363–371. 10.1016/j.neuron.2010.06.033 20696375

[B72] Huyck C. R. Passmore P. J. (2013). A review of cell assemblies. *Biol. Cybern.* 107 263–288. 10.1007/s00422-013-0555-5 23559034

[B73] Innis N. K. (2003). “William McDougall: “A major tragedy”?,” in *Portraits of Pioneers in Psychology*, eds Kimble G. A. Wertheimer M. (Washington DC: Psychology Press).

[B74] James W. (1880). The association of ideas. *Popular Sci. Monthly* 16 577–593.

[B75] James W. (1890). *The Principles of Psychology.* New York, N Y: Henry Holt & Co. [Reprinted in 1950 by Dover Publications, New York.].

[B76] Josselyn S. A. Köhler S. Frankland P. W. (2015). Finding the engram. *Nat. Rev. Neurosci.* 16 521–534. 10.1038/nrn4000 26289572

[B77] Josselyn S. A. Köhler S. Frankland P. W. (2017). Heroes of the Engram. *J. Neurosci.* 37 4647–4657. 10.1523/JNEUROSCI.0056-17.2017 28469009 PMC6596490

[B78] Keck T. Toyoizumi T. Chen L. Doiron B. Feldman D. E. Fox K. (2017). Integrating Hebbian and homeostatic plasticity: the current state of the field and future research directions. *Phil. Trans Roy. Soc. Lond. B* 372:20160158. 10.1098/rstb.2016.0158 28093552 PMC5247590

[B79] Koffka K. (1935). *Principles of Gestalt Psychology.* London: Routledge & Kegan Paul Ltd.

[B80] Köhler W. (1947). *Gestalt Psychology: An Introduction to New Concepts in Modern Psychology.* New York, NY: Liveright.

[B81] Kolb B. (2003). The impact of the Hebbian learning rule on research in behavioural neuroscience. *Canadian Psychol.* 44 14–16. 10.1037/h0085813

[B82] Konorski J. (1948). *Conditioned Reflexes and Neuron Organization.* Cambridge: Cambridge University Press.

[B83] Kruger L. Otis T. S. (2007). Whither withered Golgi? a retrospective evaluation of reticularist and synaptic constructs. *Brain Res. Bull.* 72 201–207. 10.1016/j.brainresbull.2006.11.016 17452282

[B84] Langfeld H. S. (1940). Professor McDougall’s contributions to the science of psychology. *Br. J. Psychol.* 31 107–114.

[B85] Langille J. J. Brown R. E. (2018). The synaptic theory of memory: a historical survey and reconciliation of recent opposition. *Front. Sys. Neurosci.* 12:52. 10.3389/fnsys.2018.00052 30416432 PMC6212519

[B86] Langille J. J. Gallistel C. R. (2020). Locating the engram: should we look for plastic synapses or information-storing molecules? *Neurobio. Learn. Mem.* 169:107164. 10.1016/j.nlm.2020.107164 31945459

[B87] Langley J. N. (1896). On the nerve cell connection of the splanchnic nerve fibres. *J. Physiol.* 20 223–246. 10.1113/jphysiol.1896.sp000620 16992357 PMC1512963

[B88] Langley J. N. (1905). On the reaction of cells and of nerve-endings to certain poisons, chiefly as regards the reaction of striated muscle to nicotine and to curari. *J. Physiol.* 33 374–413. 10.1113/jphysiol.1905.sp001128 16992819 PMC1465797

[B89] Lashley K. S. (1924). Studies of cerebral function in learning VI. the theory that synaptic resistance is reduced by the passage of the nerve impulse. *Psychol. Rev.* 31 369–375. 10.1037/h0070668

[B90] Lashley K. S. (1929). *Brain Mechanisms and Intelligence: A Quantitative Study of Injuries to the Brain.* Chicago, ILL: University of Chicago Press. 10.1037/10017-000

[B91] Lashley K. S. (1934). “Learning III. nervous mechanisms in learning,” in *A Handbook of General Experimental Psychology*, ed. Murchison C. (New York, NY: Russell and Russell), 456–496.

[B92] Lechner H. A. Squire L. R. Byrne J. H. (1999). 100 years of consolidation–remembering Müller and Pilzecker. *Learn. Memory* 6 77–87. 10327233

[B93] Li M. Liu J. Tsien J. Z. (2016). Theory of connectivity: nature and nurture of cell assemblies and cognitive computation. *Front. Neural Circuits* 10:34. 10.3389/fncir.2016.00034 27199674 PMC4850152

[B94] Lisman J. Cooper K. Sehgal M. Silva A. J. (2018). Memory formation depends on both synapse-specific modifications of synaptic strength and cell-specific increases in excitability. *Nat. Neurosci.* 21 309–314. 10.1038/s41593-018-0076-6 29434376 PMC5915620

[B95] Logan C. A. (2015). Engrams and biological regulation: what was “wrong” with organic memory? *Memory Stud.* 8 407–421. 10.1177/1750698015575189

[B96] López-Muñoz F. Alamo C. (2009). Historical evolution of the neurotransmission concept. *J. Neural Trans.* 116 515–533. 10.1007/s00702-009-0213-1 19350218

[B97] Lugaro E. (1909). *Modern Problems in Psychiatry.* Trans. D. Orr and E. G. Rows Manchester: The University Press.

[B98] Magee J. C. Grienberger C. (2020). Synaptic plasticity forms and functions. *Ann. Rev. Neurosci.* 43 95–117. 10.1146/annurev-neuro-090919-022842 32075520

[B99] McDougall W. (1898a). A contribution towards an improvement in psychological method. (I.). *Mind* 7 15–33.

[B100] McDougall W. (1898b). A contribution towards an improvement in psychological method. (II.). *Mind* 7 159–178.

[B101] McDougall W. (1898c). A contribution towards an improvement in psychological method. (III.). *Mind* 7 364–387.

[B102] McDougall W. (1901a). On the seat of the psycho-physical processes. *Brain* 24 579–630. 10.1093/brain/24.4.579

[B103] McDougall W. (1901b). Some new observations in support of Thomas Young’s theory of light- and colour-vision. *Mind* 10 347–382. 10.1093/mind/X.1.347

[B104] McDougall W. (1902). The physiological factors of the attention-process (I.). *Mind* 11 316–351. 10.1093/mind/XI.1.316

[B105] McDougall W. (1903a). The nature of inhibitory impulses within the nervous system. *Brain* 26 153–191. 10.1093/brain/26.2.153

[B106] McDougall W. (1903b). The physiological factors of the attention-process (II.). *Mind* 12 289–302.

[B107] McDougall W. (1903c). The physiological factors of the attention-process (III.). *Mind* 12 473–488.

[B108] McDougall W. (1905). *Primer of Physiological Psychology.* London: J.M. Dent.

[B109] McDougall W. (1906). Physiological factors of the attention process (IV.). *Mind* 15 329–359. 10.1093/mind/XV.59.329

[B110] McDougall W. (1908a). *An Introduction to Social Psychology.* London: Methuen.

[B111] McDougall A. (1908b). The physical basis of mental dissociation. *Br. Med. J.* 2 1124–1126.

[B112] McDougall W. (1908c). The state of the brain during hypnosis. *Brain* 31 242–258. 10.1093/brain/31.2.242

[B113] McDougall W. (1909). The conditions of fatigue in the nervous system. *Brain* 32 256–268. 10.1093/brain/32.3.256

[B114] McDougall W. (1912). *Psychology: The Study of Behaviour.* New York, NY: Henry Holt and Co. 10.1037/10542-000

[B115] McDougall W. (1920). *The Group Mind: a Sketch of the Principles of Collective Psychology with Some Attempt to Apply them to the Interpretation of National Life and Character.* New York, NY: Putman.

[B116] McDougall W. (1923). *An Outline of Psychology.* London: Methuen.

[B117] McDougall W. (1926a). *An Outline of Abnormal Psychology.* London: Methuen.

[B118] McDougall W. (1926b). The hypothesis of inhibition by drainage. *Psychol. Rev.* 33 370–374. 10.1037/h0073586

[B119] McDougall W. (1927). *Character and the Conduct of Life: Practical Psychology for Everyman.* London: Methuen.

[B120] McDougall W. (1929). The bearing of Professor Pavlov’s work on the problem of inhibition. *J. Gen. Psychol.* 2 231–262. 10.1080/00221309.1929.9918061

[B121] McDougall W. (1930). “William McDougall,” in *A History of Psychology in Autobiography*, ed. Murchison C. (Clark University Press: Russell & Russell), 191–223. 10.1037/11401-008

[B122] Milner P. M. (1957). The cell assembly: mark II. *Psychol. Rev.* 64 242–252. 13453608 10.1037/h0042287

[B123] Milner P. M. (2003). A brief history of the Hebbian learning rule. *Can. Psychol.* 44, 5–9. 10.1037/h0085817

[B124] Modlin I. M. Kidd M. (2001). Ernest Starling and the discovery of secretin. *J. Clin. Gastroenterol.* 32 187–192. 10.1097/00004836-200103000-00001 11246341

[B125] Morgan C. T. (1943). *Physiological Psychology.* New York, NY: McGraw-Hill.

[B126] Mott F. W. (1904). A lecture on the cerebro-spinal fluid in relation to disease of the nervous system. *Br. Med. J.* 2 1554–1560.

[B127] Mott F. W. (1907). Reviews. the integrative action of the nervous system. *Br. Med. J.* 1 567–572.

[B128] Mott F. W. Halliburton W. D. (1899). VII. the physiological action of choline and neurine. *Phil. Trans. Roy. Soc. London B.* 191 211–267. 10.1098/rstb.1899.0007 PMC2463419 20758460

[B129] Müller G. E. Pilzecker A. (1900). Experimentelle Beiträge zur Lehre vom Gedächtnis. *Zeitschrift Psychol. Physiol. Sinnesorgans Ergãnzungsband* 1 1–300.

[B130] Nadel L. Maurer A. P. (2020). Recalling Lashley and reconsolidating Hebb. *Hippocampus* 30 776–793. 10.1002/hipo.23027 30216593 PMC6417981

[B131] Pannese E. (1999). The Golgi Stain: invention, diffusion and impact on neurosciences. *J. Hist. Neurosci.* 8 132–140. 10.1076/jhin.8.2.132.1847 11624294

[B132] Pavlov I. P. (1903). “The problem of the study of higher nervous activity and the ways of its experimental solution,” in *Proceedings of the Military Medical Academy.* (New York, NY).

[B133] Pavlov I. P. (1906). The scientific investigation of the psychical faculties or processes in the higher animals. *Lancet* 4336 911–915. 10.1126/science.24.620.613 17771162

[B134] Piccolino M. (1998). Animal electricity and the birth of electrophysiology: the legacy of Luigi Galvani. *Brain Res. Bull.* 46 381–407. 10.1016/s0361-9230(98)00026-4 9739001

[B135] Pickworth F. A. (1938). A new outlook on the physiology and pathology of mental and emotional states. *Br. Med. J.* 1 265–294. 10.1136/bmj.1.4022.265 20781220 PMC2085645

[B136] Poo M. M. Pignatelli M. Ryan T. J. Tonegawa S. Bonhoeffer T. Martin K. C. (2016). What is memory? the present state of the engram. *BMC Biol.* 14:40. 10.1186/s12915-016-0261-6 27197636 PMC4874022

[B137] Quick T. (2017). Disciplining physiological psychology: cinematographs as epistemic devices in the work of Henri Bergson and Charles Scott Sherrington. *Sci. Context* 30 423–474. 10.1017/S0269889717000254 29417917

[B138] Raviola E. Mazzarello P. (2011). The diffuse nervous network of Camillo Golgi: facts and fiction. *Brain Res. Rev.* 66 75–82. 10.1016/j.brainresrev.2010.09.005 20840856

[B139] Rhine J. B. McDougall W. (1933). Third report on a Lamarckian experiment. *Br. J. Psychol.* 24 213–235.

[B140] Rhine J. B. McDougall W. (1934). *Extra Sensory Perception.* Boston MA: Boston Society for Psychic Research.

[B141] Ribatti D. (2018). An historical note on the cell theory. *Exp. Cell Res.* 364 1–4. 10.1016/j.yexcr.2018.01.038 29391153

[B142] Robins S. K. (2018). Memory and optogenetic intervention: separating the Engram from the Ecphory. *Philos. Sci.* 85 1078–1089.

[B143] Romero R. R. (2011). Marcello Malpighi (1628-1694), founder of microanatomy. *Int. J. Morphol.* 29 399–402. 10.4067/S0717-95022011000200015 27315006

[B144] Sakurai Y. Osako Y. Tanisumi Y. Ishihara E. Hirokawa J. Manabe H. (2018). Multiple approaches to the investigation of cell assembly in memory research-present and future. *Front. Sys. Neurosci.* 12:21. 10.3389/fnsys.2018.00021 29887797 PMC5980992

[B145] Schacter D. L. (2001). *Forgotten Ideas, Neglected Pioneers: Richard Semon and the Story of Memory.* New York, NY: Routledge.

[B146] Schacter D. L. Eich J. E. Tulving E. (1978). Richard Semon’s theory of memory. *J. Verbal Learn. Verbal Behav.* 17 721–743.

[B147] Schäfer E. A. (1900). *Text-book of Physiology.* Edinburgh and London: Young J. Pentland. 10.5962/bhl.title.29723

[B148] Sejnowski T. J. (2003). The once and future Hebb synapse. *Canadian Psychol.* 44 17–20. 10.1037/h0085814

[B149] Semon R. (1921). *The Mneme.* London: Allen & Unwin. (Originally published in German in 1904). (Translated into English by Louis Simon).

[B150] Semon R. (1923). *Mnemic Psychology.* London: Allen & Unwin. (Translated from the German by Bella Duffy).

[B151] Semon R. W. (1912). *Das Problem der Vererbung “erworbener Eigenschaften”.* Leipzig: Verlag von Wilhelm Engelmann.

[B152] Serrano-Castro P. J. Garcia-Torrecillas J. M. (2012). Cajal’s first steps in scientific research. *Neuroscience* 217 1–5. 10.1016/j.neuroscience.2012.05.008 22588002

[B153] Sharpey-Schafer E. (1927). History of the Physiological Society during its first fifty years, 1876-1926. *J. Physiol.* 64 (3 Suppl.), 1–77.16993897

[B154] Sharpey-Schafer E. (1932). Observations on the history of physiology in great Britain during the last hundred years. *Br. Med. J.* 2 781–783. 20777136 10.1136/bmj.2.3747.781 PMC2521890

[B155] Shepherd G. M. (2010). *Creating Modern Neuroscience: the Revolutionary 1950s.* Oxford: Oxford University Press, 10.1093/acprof:oso/9780195391503.001.0001

[B156] Shepherd G. M. (2016). *Foundations of the Neuron Doctrine, 25th Anniversary Edition.* Oxford: Oxford University Press.

[B157] Shepherd G. M. Greer C. A. Mazzarello P. Sassoè-Pognetto M. (2011). The first images of nerve cells: golgi on the olfactory bulb 1875. *Brain Res. Rev.* 6 92–105. 10.1016/j.brainresrev.2010.09.009 20940020 PMC4465565

[B158] Sherrington C. S. (1897). Croonian lecture,- the mammalian spinal cord as an organ of reflex action [Abstract]. *Proc. Roy. Soc. Lond.* 61 220–221. 10.1098/rspl.1897.0025

[B159] Sherrington C. S. (1898). “The activity of the nervous centers which correlate antagonistic muscles in the limbs,” in *Proceedings of the Report of the 67th Meeting of the British Association for the Advancement of Science, held at Toronto in August* 1897, (London: John Murry).

[B160] Sherrington C. S. (1900). “The spinal cord,” in *Text-book of Physiology*, ed. Schäfer E. A. (Edinburgh: Pentland).

[B161] Sherrington C. S. (1905). “Correlation of reflexes and the principle of the common path,” in *Proceedings of the Report of the 74th Meeting of the British Association for the Advancement of Science, held at Cambridge in August* 1904, (London: John Murray), 728–741.

[B162] Sherrington C. S. (1906a). Observations on the scratch-reflex in the spinal dog. *J. Physiol.* 34 1–50. 10.1113/jphysiol.1906.sp001139 16992835 PMC1465804

[B163] Sherrington C. S. (1906b). *The Integrative Action of the Nervous System.* New Haven: Yale University Press.

[B164] Sherrington C. S. Grünbaum A. S. (1902). Observations on the physiology of the cerebral cortex of some of the higher apes. (Preliminary communication). *Proc. Roy. Soc. London.* 69 206–209. 10.1098/rspl.1901.0100

[B165] Sherrington C. S. McDougall W. MacDonald J. S. Lawson H. S. (1911). “Mental and muscular fatigue,” in *Proceedings of the Report of the committee. Report of the 18th Meeting of the British Association for the Advancement of Science, Sheffield: 1910.* (London: John Murray).

[B166] Sherrington C. S. McDougall W. MacDonald J. S. Lawson H. S. Chapman J. E. (1912). “Mental and muscular fatigue,” in *Proceedings of the Report of the Committee. Report of the 18th Meeting of the British Association for the Advancement of Science, Portsmouth 1911.* (London: John Murray).

[B167] Smith C. U. (1996). Sherrington’s legacy: evolution of the synapse concept, 1890s-1990s. *J. Hist. Neurosci.* 5 43–55. 10.1080/09647049609525650 11619033

[B168] Sourkes T. L. (2009). Acetylcholine: from vagusstoff to cerebral neurotransmitter. *J. Hist. Neurosci.* 18 47–58. 10.1080/09647040701632305 19160113

[B169] Spatz H. C. (1996). Hebb’s concept of synaptic plasticity and neuronal cell assemblies. *Behav. Brain Res.* 78 3–7. 10.1016/0166-4328(95)00221-9 8793031

[B170] Spiering M. J. (2018). The discovery of GABA in the brain. *J. Biol. Chem.* 293 19159–19160. 10.1074/jbc.CL118.006591 30530855 PMC6295731

[B171] Stahnisch F. W. (2015). Joseph von Gerlach (1820-1896). *J. Neurol.* 262 1397–1399. 10.1007/s00415-015-7735-2 25893254

[B172] Sweatt J. D. (2016). Neural plasticity and behavior - sixty years of conceptual advances. *J. Neurochem.* 139 (Suppl. 2), 179–199. 10.1111/jnc.13580 26875778

[B173] Takamiya S. Yuki S. Hirokawa J. Manabe H. Sakurai Y. (2020). Dynamics of memory engrams. *Neurosci. Res.* 153 22–26. 10.1016/j.neures.2019.03.005 30940458

[B174] Tansey E. M. (1991). Chemical neurotransmission in the autonomic nervous system: sir Henry Dale and acetylcholine. *Clin. Auton. Res* 1 63–72. 10.1007/BF01826060 1668257

[B175] Tansey E. M. (1997). Not Committing Barbarisms: Sherrington and the Synapse. *Brain Res. Bull.* 44 211–212. 9323432 10.1016/s0361-9230(97)00312-2

[B176] Tanzi E. (1893). I fatti e le induzioni dell’odierna istologia del sistema nervoso. *Rivista Sperimentale di Freniatria* 19 419–472.

[B177] Taoufik E. Kouroupi G. Zygogianni O. Matsas R. (2018). Synaptic dysfunction in neurodegenerative and neurodevelopmental diseases: an overview of induced pluripotent stem-cell-based disease models. *Open Biol.* 8:180138. 10.1098/rsob.180138 30185603 PMC6170506

[B178] Thompson R. F. (1976). The search for the engram. *Am. Psychol.* 31 209–227. 10.1037//0003-066x.31.3.209 766675

[B179] Tizard B. (1959). Theories of brain localization from Flourens to Lashley. *Med. Hist.* 3 132–145. 10.1017/s0025727300024418 13643147 PMC1034464

[B180] Todman D. (2008). Henry dale and the discovery of chemical synaptic transmission. *Eur. Neurol.* 60 162–164. 10.1159/000145336 18645249

[B181] Triarhou L. C. del Cerro M. (1985). Freud’s contribution to neuroanatomy. *Arch. Neurol.* 42 282–287. 10.1001/archneur.1985.04060030104017 3883963

[B182] Tyler K. L. Malessa R. (2000). The Goltz-Ferrier debates and the triumph of cerebral localizationalist theory. *Neurology* 55 1015–1024. 10.1212/wnl.55.7.1015 11061261

[B183] Valentine E. R. (1999). The founding of the Psychological Laboratory, University College London: “Dear Galton…Yours truly, J Sully.” *Hist. Psychol.* 2, 204–218. 10.1037/1093-4510.2.3.204 11623922

[B184] Valenstein E. S. (2002). The discovery of chemical neurotransmitters. *Brain Cogn.* 49 73–95. 10.1006/brcg.2001.1487 12027394

[B185] Waldeyer W. (1891). Ueber einige neuere Forschungen im Gebiete der Anatomie des Centralnervensystems. *Dtsch. Med. Wochenschr* 17 1213–1218.

[B186] Ward J. (1886). “Psychology,” in *Encyclopaedia Britannica*, 9th Edn, (Edinburgh: Black), 37–85.

[B187] Watson J. B. McDougall W. (1929). The Battle of Behaviourism: An Exposition and an Exposure. New York: W. W. Norton & Co.

[B188] Weyers W. (2020). Joseph von Gerlach-A reminder of one of the pioneers of histochemistry on the occasion of his 200th birthday. *Am. J. Dermatopathol.* 42 731–738. 10.1097/DAD.0000000000001739 32675471

[B189] White H. W. (1908). “Neurorrheuma” or “Neurokyme”. *Br. Med. J.* 2:1216.

[B190] Wilson J. D. (1944). George charles moore smith,1858-1940. *Proc. Br. Acad.* 30 361–377.

[B191] Winkelmann A. (2007). Wilhelm von Waldeyer-Hartz (1836-1921): an anatomist who left his mark. *Clin. Anat.* 20 231–234. 10.1002/ca.20400 17072873

[B192] Wolpert L. (1995). Evolution of the cell theory. *Phil. Trans. Roy. Soc. B.* 349 227–233. 10.1098/rstb.1995.0106 8577831

[B193] Wu C. Q. (2012). A neurobiologically based two-stage model for human color vision. *Proc. SPIE* 8291:829110. 10.1117/12.909692

[B194] Yates F. A. (1966). *The Art of Memory.* London: Routledge & Kegan Paul.

[B195] Zigmond M. J. (1999). Otto Loewi and the demonstration of chemical neurotransmission. *Brain Res. Bull.* 50 347–348. 10.1016/s0361-9230(99)00099-4 10643430

[B196] Zola-Morgan S. (1995). Localization of brain function: the legacy of Franz Joseph Gall (1758-1828). *Ann. Rev. Neurosci.* 18 359–383. 10.1146/annurev.ne.18.030195.002043 7605066

